# Ursodeoxycholic acid induces sarcopenia associated with decreased protein synthesis and autophagic flux

**DOI:** 10.1186/s40659-023-00431-8

**Published:** 2023-05-27

**Authors:** Josué Orozco-Aguilar, Franco Tacchi, Francisco Aguirre, Mayalen Valero-Breton, Mauricio Castro-Sepulveda, Felipe Simon, Claudio Cabello-Verrugio

**Affiliations:** 1 Laboratory of Muscle Pathology, Fragility and Aging, Faculty of Life Sciences, Universidad Andres Bello, Santiago, Chile; 2 Millennium Institute On Immunology and Immunotherapy, Faculty of Life Sciences, Universidad Andres Bello, Santiago, Chile; 3 Facultad de Farmacia, Universidad de Costa Rica, San José, Costa Rica; 4 Exercise Physiology and Metabolism Laboratory, School of Kinesiology, Faculty of Medicine, Finis Terrae University, Santiago, Chile; 5 Laboratory of Integrative Physiopathology, Faculty of Life Sciences, Universidad Andres Bello, Santiago, Chile; 6 Millennium Nucleus of Ion Channel-Associated Diseases (MiNICAD), Universidad de Chile, Santiago, Chile

**Keywords:** Sarcopenia, Ursodeoxycholic acid, Autophagic flux, Protein synthesis, Bile acids

## Abstract

**Background:**

Skeletal muscle generates force and movements and maintains posture. Under pathological conditions, muscle fibers suffer an imbalance in protein synthesis/degradation. This event causes muscle mass loss and decreased strength and muscle function, a syndrome known as sarcopenia. Recently, our laboratory described secondary sarcopenia in a chronic cholestatic liver disease (CCLD) mouse model. Interestingly, the administration of ursodeoxycholic acid (UDCA), a hydrophilic bile acid, is an effective therapy for cholestatic hepatic alterations. However, the effect of UDCA on skeletal muscle mass and functionality has never been evaluated, nor the possible involved mechanisms.

**Methods:**

We assessed the ability of UDCA to generate sarcopenia in C57BL6 mice and develop a sarcopenic-like phenotype in C_2_C_12_ myotubes and isolated muscle fibers. In mice, we measured muscle strength by a grip strength test, muscle mass by bioimpedance and mass for specific muscles, and physical function by a treadmill test. We also detected the fiber’s diameter and content of sarcomeric proteins. In C_2_C_12_ myotubes and/or isolated muscle fibers, we determined the diameter and troponin I level to validate the cellular effect. Moreover, to evaluate possible mechanisms, we detected puromycin incorporation, p70S6K, and 4EBP1 to evaluate protein synthesis and ULK1, LC3 I, and II protein levels to determine autophagic flux. The mitophagosome-like structures were detected by transmission electron microscopy.

**Results:**

UDCA induced sarcopenia in healthy mice, evidenced by decreased strength, muscle mass, and physical function, with a decline in the fiber’s diameter and the troponin I protein levels. In the C_2_C_12_ myotubes, we observed that UDCA caused a reduction in the diameter and content of MHC, troponin I, puromycin incorporation, and phosphorylated forms of p70S6K and 4EBP1. Further, we detected increased levels of phosphorylated ULK1, the LC3II/LC3I ratio, and the number of mitophagosome-like structures. These data suggest that UDCA induces a sarcopenic-like phenotype with decreased protein synthesis and autophagic flux.

**Conclusions:**

Our results indicate that UDCA induces sarcopenia in mice and sarcopenic-like features in C_2_C_12_ myotubes and/or isolated muscle fibers concomitantly with decreased protein synthesis and alterations in autophagic flux.

**Supplementary Information:**

The online version contains supplementary material available at 10.1186/s40659-023-00431-8.

## Introduction

Chronic cholestatic liver diseases (CCLD) are a group of progressive alterations affecting the liver, beginning with bile conduct obstruction leading to hepatic dysfunction, such as liver fibrosis, steatosis, and cirrhosis [1]. Patients with CCLD develop secondary sarcopenia due to hepatic dysfunction [2, 3]. CCLD-associated sarcopenia has a high prevalence, reaching 40–70% of patients with CCLD [4]. Ultimately, sarcopenia during CCLD is relevant because it is considered a predictor of mortality [5].

Sarcopenia is characterized by decreased muscle strength and mass and a decline in physical activity [6]. Several features are observed in sarcopenic muscle, such as unbalanced sarcomeric proteostasis, oxidative stress, deregulation of autophagy, myonuclear apoptosis, and mitochondrial dysfunction [7, 8]. We have recently described a mouse model of CCLD-induced sarcopenia, in which mice with a hepatic injury induced by a hepatotoxin developed sarcopenia [9]. Muscle alterations, including decreased mass, strength, and physical activity, together with reduced content of myofibrillar proteins (troponin I and myosin heavy chain (MHC)), increased ubiquitin–proteasome system (UPS) activity (precisely, the expression of E3 ligases MuRF-1 and atrogin-1), oxidative stress and alteration in myonuclear apoptosis [9–11]. Indeed, this model is characterized by increased serum bile acids (BA). The importance of cholestasis in this model was evaluated by the absence of TGR5, a BA membrane receptor sufficient to prevent sarcopenia [12]. Interestingly, many features of this murine model of CCLD-induced sarcopenia are reproducible by treating myotubes or isolated muscle fibers with bile acids such as cholic acid (CA) and deoxycholic acid (DCA) [13, 14].

Ursodeoxycholic acid (UDCA) is formed by epimerization of the primary BA chenodeoxycholic acid in the gut by intestinal bacteria and is the most hydrophilic BA. Its polar structure is characterized by lower toxicity than other BA [15–17]. Actually, UDCA is a pharmacological option to treat cholestatic diseases, such as primary biliary cholangitis, primary sclerosing cholangitis or intrahepatic cholestasis of pregnancy [18]. Besides the hepatoprotective role, UDCA has also been assessed in treating extrahepatic diseases. Thus, UDCA has improved pre-clinical models of neurodegenerative diseases such as Parkinson’s, Huntington’s, and Alzheimer’s by preventing mitochondrial dysfunction and apoptosis and restoring cognitive and locomotor functions. UDCA has shown anti-inflammatory, immunosuppressant, and cytoprotective actions in preclinical models of nonalcoholic fatty liver disease (NAFLD), diabetes, obesity [19–22], myocardial infarction [23], acute kidney injury [24, 25], asthma [26], arthritis [27], diabetic retinitis and retinitis pigmentosa [20, 28, 29].

Although the chemical nature of UDCA suggests a possible interaction between this BA and TGR5, data regarding TGR5 activation that depends on UDCA is conflicted. Some reports have detailed UDCA as a central agonist of TGR5, while other investigations, mainly with cells overexpressing TGR5, did not show UDCA agonism. These data suggest that UDCA could activate TGR5, depending on the cell context [30–34]. Interestingly, it is not known whether a possible effect of UDCA in skeletal muscle depends on the TGR5 receptor.

Considering the beneficial features of UDCA in treating hepatic dysfunctions, in the present study, we evaluated the effect of UDCA administration on the skeletal muscle of mice. We determined that UDCA at a dosage pharmacologically relevant induces sarcopenia in mice through a decline in muscle mass, strength, and physical activity. We observed a sarcopenic-like phenotype in skeletal muscle cells and isolated muscle fibers by decreasing the diameter and content of sarcomeric proteins. In addition, using myotubes or isolated muscle fiber cultures, we elucidated that the mechanisms involved in the sarcopenic-like phenotype are independent of the TGR5 receptor, with alterations in the protein synthesis pathways and autophagy.

## Results

### Ursodeoxycholic acid induces sarcopenia in mice

Sarcopenia is characterized by a decline in muscle mass, strength, and physical function [6]. Therefore, we evaluated the effect of UDCA on the possible generation of muscle wasting and sarcopenia in mice. Adult mice were treated with UDCA (200 mg/kg) for 6 weeks, and muscle mass was determined from bioelectrical impedance analysis (BIA) measurements. Table 1 shows that mice treated with UDCA exhibited increased fat mass (FM) and decreased total body water (TBW) and fat-free mass (FFM), suggesting a decline in the entire body muscle mass. Complementarily, we determined the mass of several muscles, such as the extensor digitorum longus (EDL), tibialis anterior (TA), gastrocnemius (GAST), soleus (SOL), and diaphragm (DIA) (Table 1). The results showed a decline in mass in all these muscles in UDCA-treated mice. However, the reduction in muscle mass was not due to changes in body weight (Table 1).

**Table 1:** Physiological parameters in adult mice treated with 200 mg/kg UDCA

Physiological parameter		Control	UDCA
FFM (g)		14.98 ± 0.94	10.21 ± 0.75*
TBW (mL)		10.97 ± 0.67	7.47 ± 0.55*
FM (g)		4.16 ± 0.45	7.14 ± 0.80*
Muscle mass (mg)	EDL	10.08 ± 0.39	7.32 ± 0.21*
	TA	42.76 ± 1.20	37.51 ± 1.05*
	GAST	109.5 ± 2.5	100.6 ± 1.4*
	SOL	9.66 ± 0.29	7.77 ± 0.20*
	DIA	37.62 ± 2.13	31.91 ± 1.53*
Body weight (g)	Initial	30.13 ± 1.9	31.31 ± 3.1
	Final	29.10 ± 1.5	30.53 ± 1.9

Data correspond to mean ± SEM (n = 5–6 animals per condition). Data for Body weight correspond to mean ± SD (n = 6 animals per condition). *p < 0.05, t-test. *DIA* diaphragm, *EDL* extensor digitorum longus, *FFM* fat-free mass, *FM* fat mass, *GAST* gastrocnemius, *SOL* soleus, *TA* tibialis anterior, *TBW* total body water, *UDCA* ursodeoxycholic acid

**Fig. 1:**
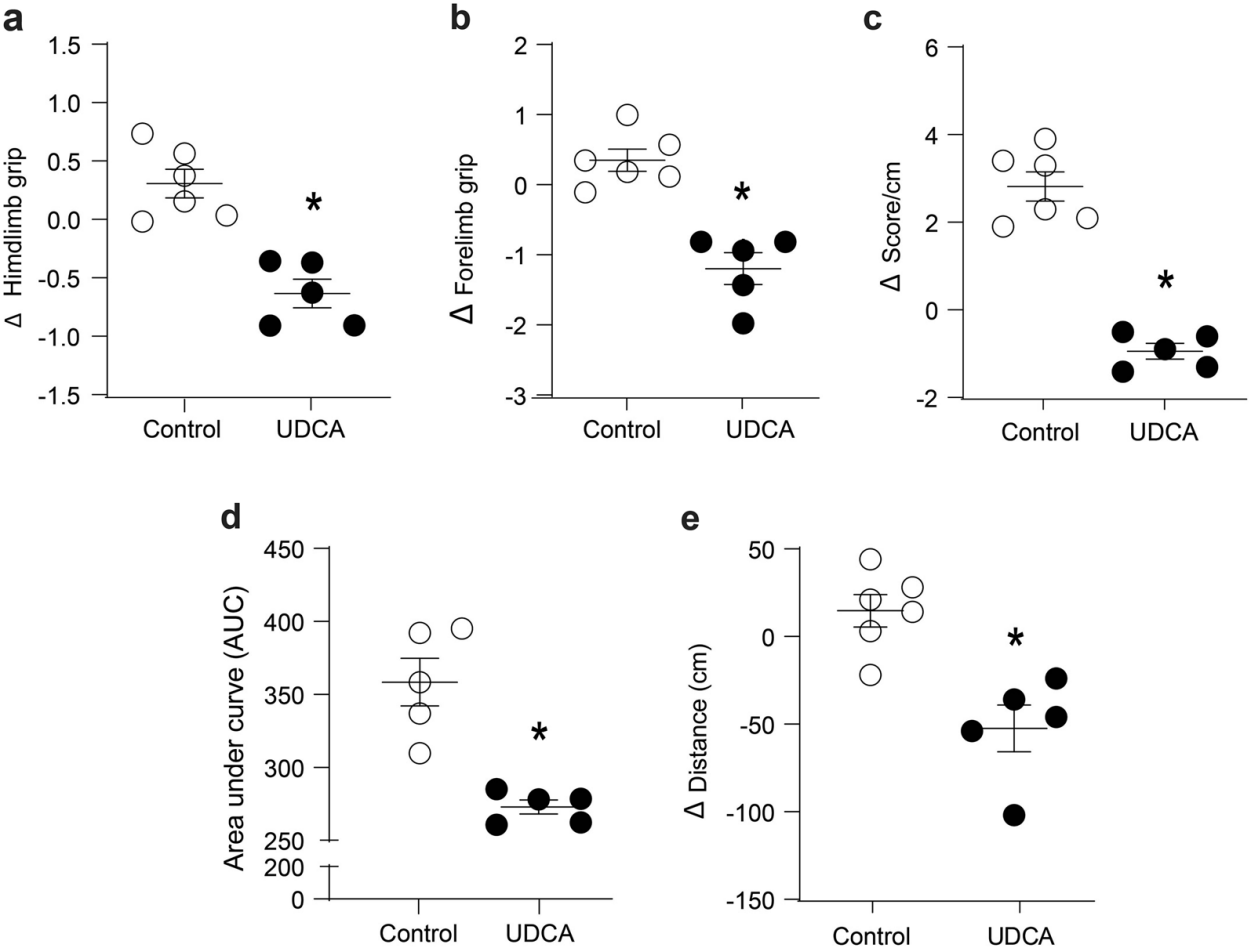
Muscle function declines in mice treated with UDCA after 6 weeks. C57BL/6 male mice were treated orally with UDCA 200 mg/kg corporal body weight for 6 weeks (1.04% NaCl pH 8.4 to the control group). Mice were monitored daily, and muscular evaluations were made at the beginning and during the sixth week. **a** The hindlimb strength was measured by a dynamometer. Values represent the difference between the initial measure before UDCA treatment and the end of treatment. **b** The forelimb strength was measured by a dynamometer. Values represent the difference between the initial measure before UDCA treatment and the end of treatment. **c** The forelimb strength was measured by a weightlifting test. Values represent the difference between the initial score normalized before UDCA treatment and the end of treatment. **d** After euthanizing, the TA muscle was dissected to evaluate the electromyography performance. The area under the curve was calculated for each animal using GraphPad Prism 8.0 software and represented individually. **e** The maximum distance reached by each animal on a treadmill was recorded. Values represent the difference between the initial distance before UDCA treatment and the end of treatment. The result shows the individual value of the subject, with the mean ± SEM for each group. (n = 5 mice per group, no paired t-test, *p < 0.05 with respect to the control group). *SEM* standard error of the mean, *UDCA* ursodeoxycholic acid

Then, we determined the effect of the UDCA on muscle strength in live mice using a grip strength test. Our results show that UDCA reduced the hindlimb strength 6 weeks after UDCA administration (Fig. 1a) compared to vehicle-treated mice (control mice). Similar effects were observed in the strength of forelimbs 6 weeks after UDCA treatment (Fig. 1b). Complementary to these results, we evaluated muscle strength through the weightlifting test. Figure 1c shows a decreased force in UDCA-treated mice. Thus, we conclude that UDCA administration can cause a decline in strength in live mice. Next, we evaluated the muscular contribution in the UDCA-induced weakness by measuring ex vivo muscle strength in TA-isolated muscles. We determined the tetanic force using electrophysiological measurements. Our results show isolated TA-generated strength was lower in UDCA than in control mice (Fig. 1d).

Finally, we evaluated the physical activity in response to UDCA administration using the incremental maximal running test. Figure 1e shows that the distance run by mice treated with UDCA was lower than the distance run by the control mice. In addition, we determined the effect of UDCA in a medium- or low-effort test, namely the rotarod and open-field tests, respectively. We observed that UDCA did not change the time that mice were able to keep going on the rotarod (Additional file 1a) or the no-forced locomotion distance in the open field test (Additional file 1b).

These data indicate that UDCA induces sarcopenia in mice by decreasing muscle mass, strength, and physical activity.

### Ursodeoxycholic acid induces morphological alterations associated with sarcopenia in skeletal muscle

**Fig. 2:**
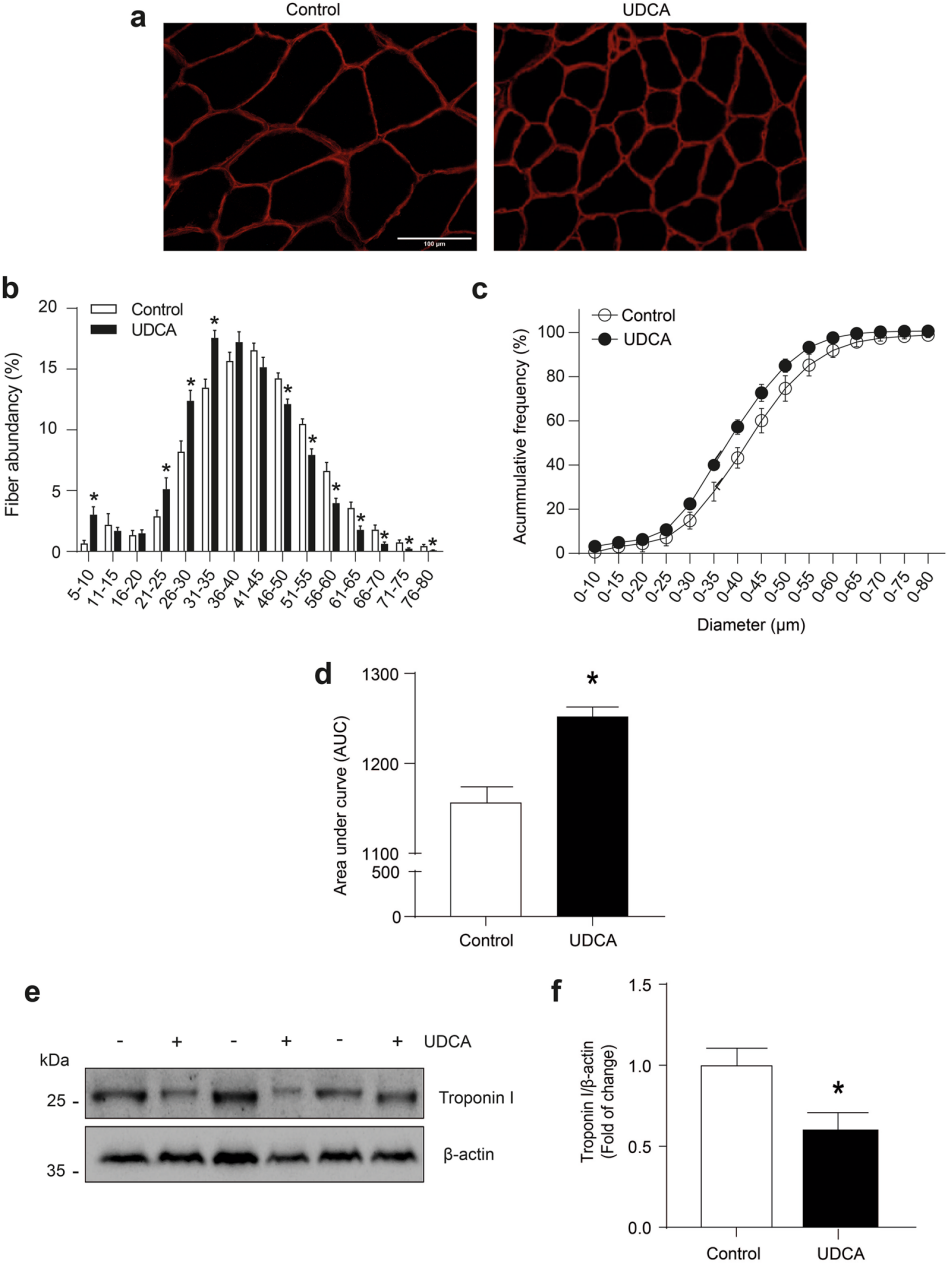
After 6 weeks, UDCA treatment diminishes the fiber diameter and troponin I level in adult mice. C57BL/6 male mice were treated orally with UDCA 200 mg/kg corporal body weight for 6 weeks (1.04% NaCl pH 8.4 to the control group). Mice were monitored daily and were euthanized during the sixth week. **a** TA muscle cross-sections were stained with laminin to delimit the sarcolemma. The scale bar indicates 100 μm. **b** The minimal Feret’s diameters were calculated using the MyoVision software. Fiber diameters were grouped from 5 to 80 μm to quantify the total fiber percentage in each group. **c** Accumulative frequency analysis to UDCA and control group were plotted. **d** The area under the curve was calculated in accumulative frequency to fiber diameters using GraphPad Prism 8.0 software. **e** TA muscle was homogenized to evaluate troponin I protein levels by western blot analysis, using β-actin as the loading control, and the molecular weights are depicted in kilodaltons (kDa). **f** Quantitative analysis for troponin I protein levels is shown. The result shows the mean ± SEM for each group (n = 5 mice per group, no paired t-test, *p < 0.05 with respect to the control group). *SEM* standard error of the mean, *UDCA* ursodeoxycholic acid

A typical pathological feature of sarcopenia is decreased fiber diameter [9, 12]. Thus, we evaluated the effect of UDCA on the distribution of fiber diameter in TA muscles, using immunofluorescent detection of laminin to delimit the muscle fibers (Fig. 2a). These data were quantified and are shown in Fig. 2b. The result indicates that UDCA generated a higher abundance of fiber with a lower diameter than control mice. The same Fig. 2b also shows that mice treated with UDCA exhibited a lower quantity of fiber with a higher diameter than control mice. Analysis of the cumulative frequency of fiber sizes reveals that the curve generated by UDCA treatment was displaced to the left, indicating a higher abundance towards small sizes than control mice (Fig. 2c). The area under the curve (AUC) was quantified from these curves. The result shows that UDCA-treated mice had an increment in the AUC compared with control mice (Fig. 2d). We also evaluated the fiber diameter in the soleus (SOL), a slow-oxidative muscle (Additional file 2a). We observed that UDCA decreased the fiber size showing a more abundance of small fibers than control mice (Additional file 2b), showing a displacement towards the left of UDCA-treated mice in the curve of accumulative frequency (Additional file 2c). This change is reflected in an increased AUC of UDCA mice compared to control mice (Additional file 2d).

Another typical feature of sarcopenia is a decline in the content of myofibrillar proteins belonging to the sarcomere, such as troponin I. Therefore, we evaluated the effect of UDCA on the levels of such proteins through western blot analysis (Fig. 2e). The densitometric analysis results show that UDCA decreased the troponin I protein levels in TA muscles (Fig. 2f).

Together, these results indicate that UDCA-induced sarcopenia in mice involves, at least, a decrease in fiber diameter and myofibrillar sarcomeric proteins.

### Ursodeoxycholic acid induces a sarcopenic-like phenotype in myotubes and single-isolated muscle fibers

**Fig. 3:**
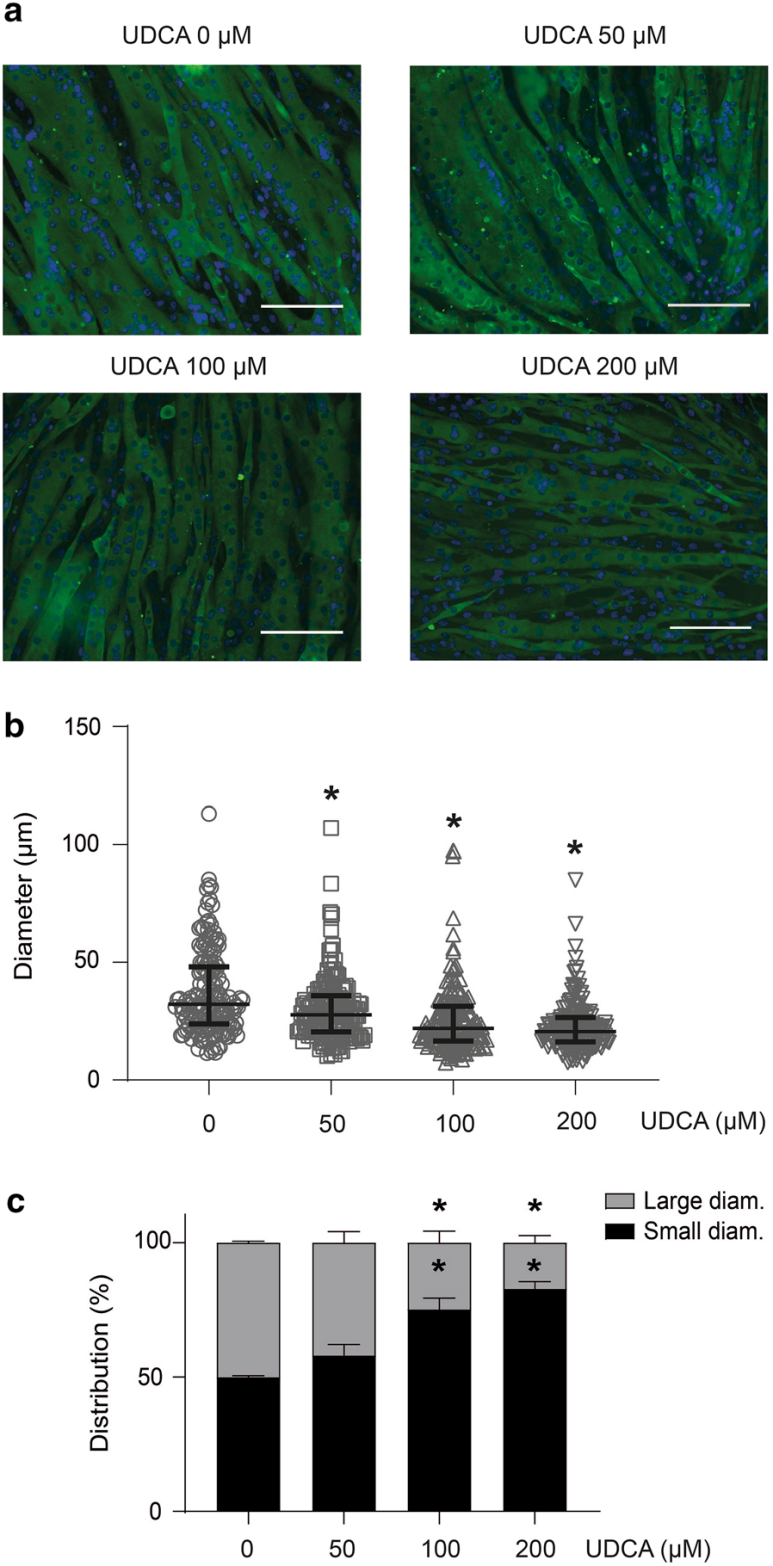
UDCA reduces the diameter of C_2_C_12_ myotubes in a dose-dependent manner. Myotubes were incubated with 0–50–100–200 μM UDCA for 72 h. **a** MHC was detected by indirect immunofluorescence and used to delimit the myotube diameter. Images were captured by fluorescence microscopy. The scale bar indicates 100 μm. **b** The diameter of the myotubes was measured using ImageJ software. The quantification was performed, and the individual values for myotube were plotted. **c** The myotubes were classified using the control group median (32.13 μm). Myotubes with large or small diameters were determined if their diameter was larger or smaller than the threshold value. The values indicate the percentage of myotubes expressed as the mean ± SEM of three independent experiments (one‐way ANOVA, post‐hoc Dunnet, *p < 0.05 with respect to the control). *ANOVA* analysis of variance, *MHC* myosin heavy chain, *SEM* standard error of the mean, *UDCA* ursodeoxycholic acid

Some features present in sarcopenic muscles can be observed in vitro using a culture of myotubes and single isolated fibers from muscles [14]. First, we administered different doses of UDCA to determine the viability of myotubes. No evidence of cytotoxicity was shown in all doses at 48 h (Additional file 3a) or under 400 μM at 72 h (Additional file 3b). Then, we assessed the dose-dependent effect of UDCA on the diameter of C_2_C_12_ myotubes. Figure 3a shows the delimited surface of myotubes revealed by MHC immunofluorescent detection with several UDCA dosages. Figure 3b shows the heterogeneous distribution of the myotube diameter in all experimental conditions. More important is the fact that UDCA decreased the average diameter in a UDCA dose-independent fashion (Fig. 3b). Further, we classified the myotubes into two categories: if the value of the myotubes’ diameter was above the median value of the control group, they were considered to have a large diameter, whereas if the myotubes’ median diameter was below the median value of the control group, they were supposed to have a small diameter. According to this classification, our results show that myotubes with low diameters increased, whereas those with high diameters decreased in a UDCA dose-dependent manner (Fig. 3c).

**Fig. 4:**
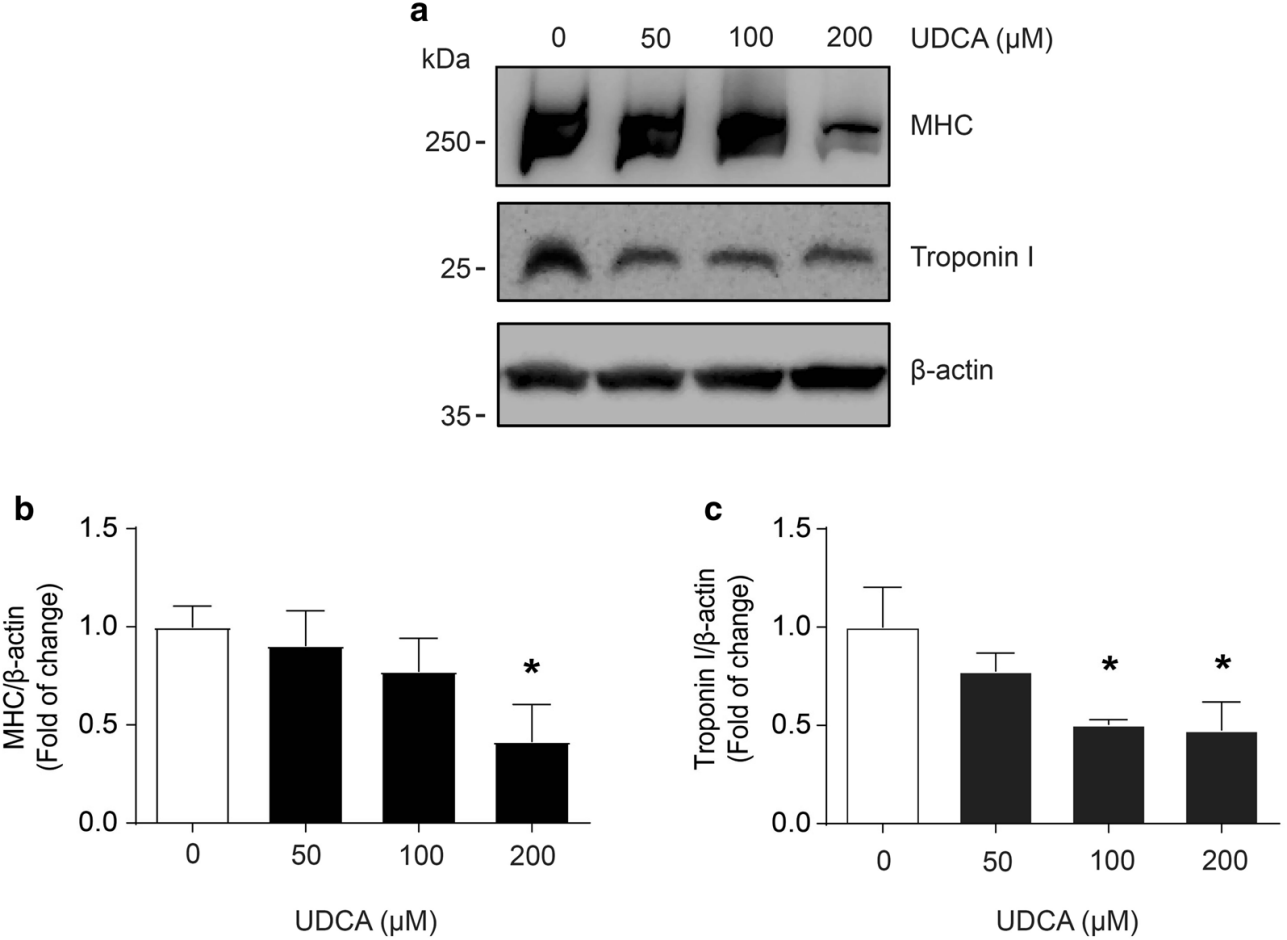
UDCA reduces sarcomeric proteins in C_2_C_12_ myotubes. C_2_C_12_ myoblasts were differentiated for 4–5 days and incubated with 0, 50, 100, 200, and 400 μM UDCA for 72 h. **a** MHC and troponin I protein levels were detected by western blot analysis, using β‐actin as the loading control. Molecular weights are shown in kDa. Densitometry analysis for (**b**) MHC and (**c**) troponin I is represented as a fold of change expressed as the mean ± SD of three independent experiments (no paired t-test, *p < 0.05 with respect to the control group). *MHC* myosin heavy chain, *SD* standard deviation, *UDCA* ursodeoxycholic acid

Another typical parameter affected in sarcopenic-like phenotype is a decrease in the content of sarcomeric proteins such as MHC and troponin I. Figure 4a shows a UDCA dose-dependent effect on MHC and troponin I protein levels revealed by western blot analysis. These data are quantified in Fig. 4b and c for MHC and troponin I, respectively. The results indicate that UDCA decreased the MHC protein levels at 200 μM, whereas UDCA decreased troponin I at 100 μM.

**Fig. 5:**
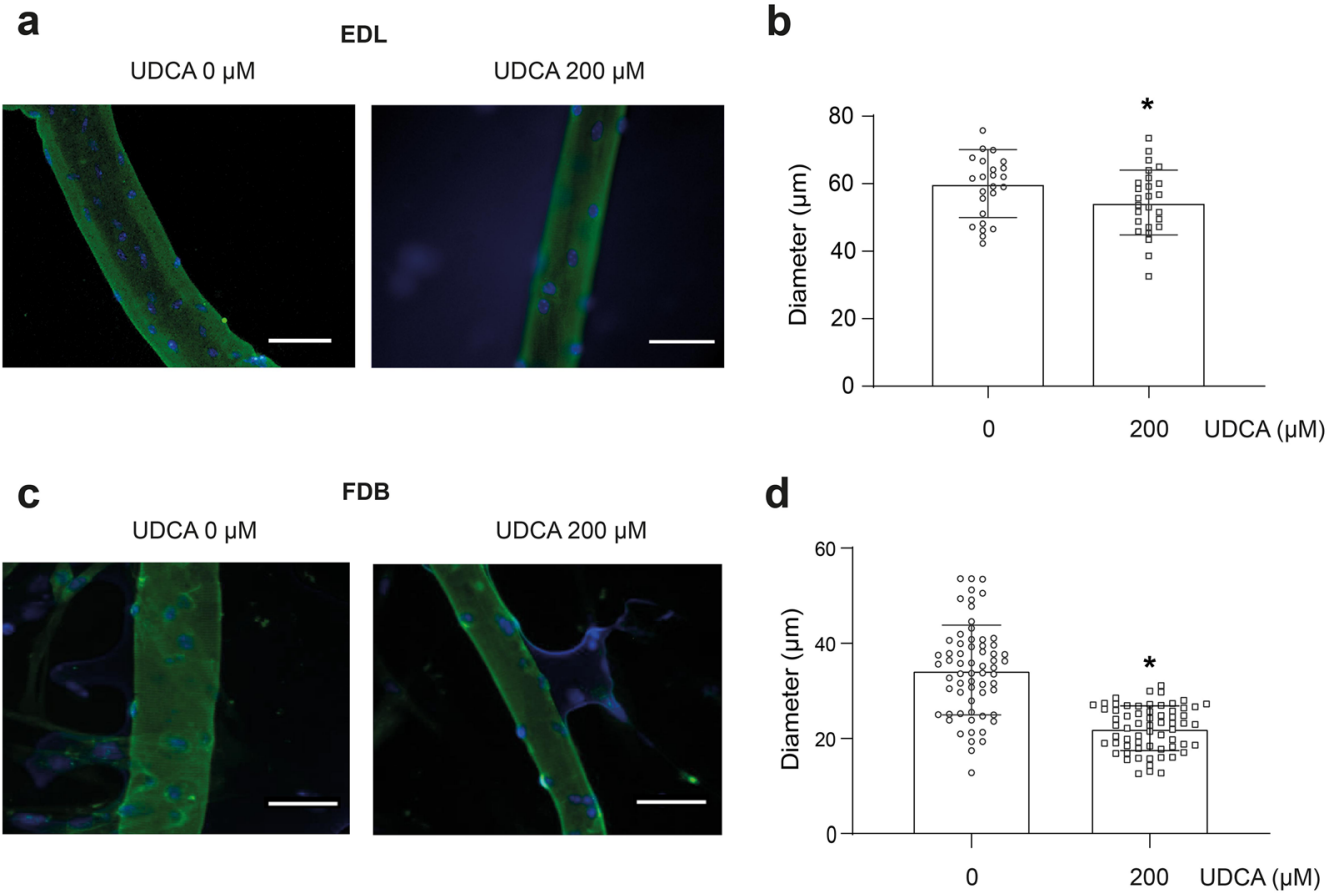
Isolated muscle fiber diameter is reduced by UDCA treatment. Muscle fibers were isolated from C57BL/6 male mice and incubated with 200 μM UDCA for 72 h. **a** Indirect immunofluorescence detected EDL muscle fiber MHC to delimit the fiber diameter. Fluorescent microscopy images were captured. The scale bar indicates 50 μm. **b** The diameter of the isolated muscle fiber was measured using ImageJ software. The quantification was performed, and the individual values for fiber were plotted. **c** FDB muscle fiber MHC was detected by indirect immunofluorescence to delimit the fiber diameter. Fluorescent microscopy images were captured. The scale bar indicates 50 μm. **d** The diameter of the isolated muscle fiber was measured using ImageJ software. The quantification was performed, and the individual values for fiber were plotted. The result shows the value of each fiber, with the mean ± SD for each group (n = 27 EDL muscle fibers, 60 FDB muscle fibers, no paired t-test with Welch’s correction, *p < 0.05 with respect to the control group). *EDL* extensor digitorum longus, *FDB* flexor digitorum brevis, *MHC* myosin heavy chain, *SD* standard deviation, *UDCA* ursodeoxycholic acid

To corroborate our data, we evaluated the effect of UDCA on the diameter of the single isolated fiber from skeletal muscles. In EDL- (Fig. 5a and b) and flexor digitorum brevis (FDB)-isolated fibers (Fig. 5c and d), a UDCA-induced decrease in the fiber diameter compared to control fibers (UDCA 0 μM) was observed.

UDCA can bind to the TGR5 receptor [33, 35]. Moreover, we have previously described that DCA and CA induce sarcopenic-like features through the TGR5 receptor [14]. Therefore, we evaluated if the sarcopenic-like phenotype induced by UDCA is mediated through the TGR5 receptor by using its inhibitor SBI-115. Additional file 4a and b show that the TGR5 inhibitor SBI-115 did not prevent the decrease in myotube diameter caused by UDCA. Similarly, this inhibitor could not prevent the reduced troponin I level induced by UDCA (Additional file 4c and d).

Our data demonstrated that UDCA induces a sarcopenic-like phenotype in myotubes and muscle fibers by a mechanism independent of the TGR5 receptor.

### Ursodeoxycholic acid reduces the protein synthesis in myotubes

**Fig. 6:**
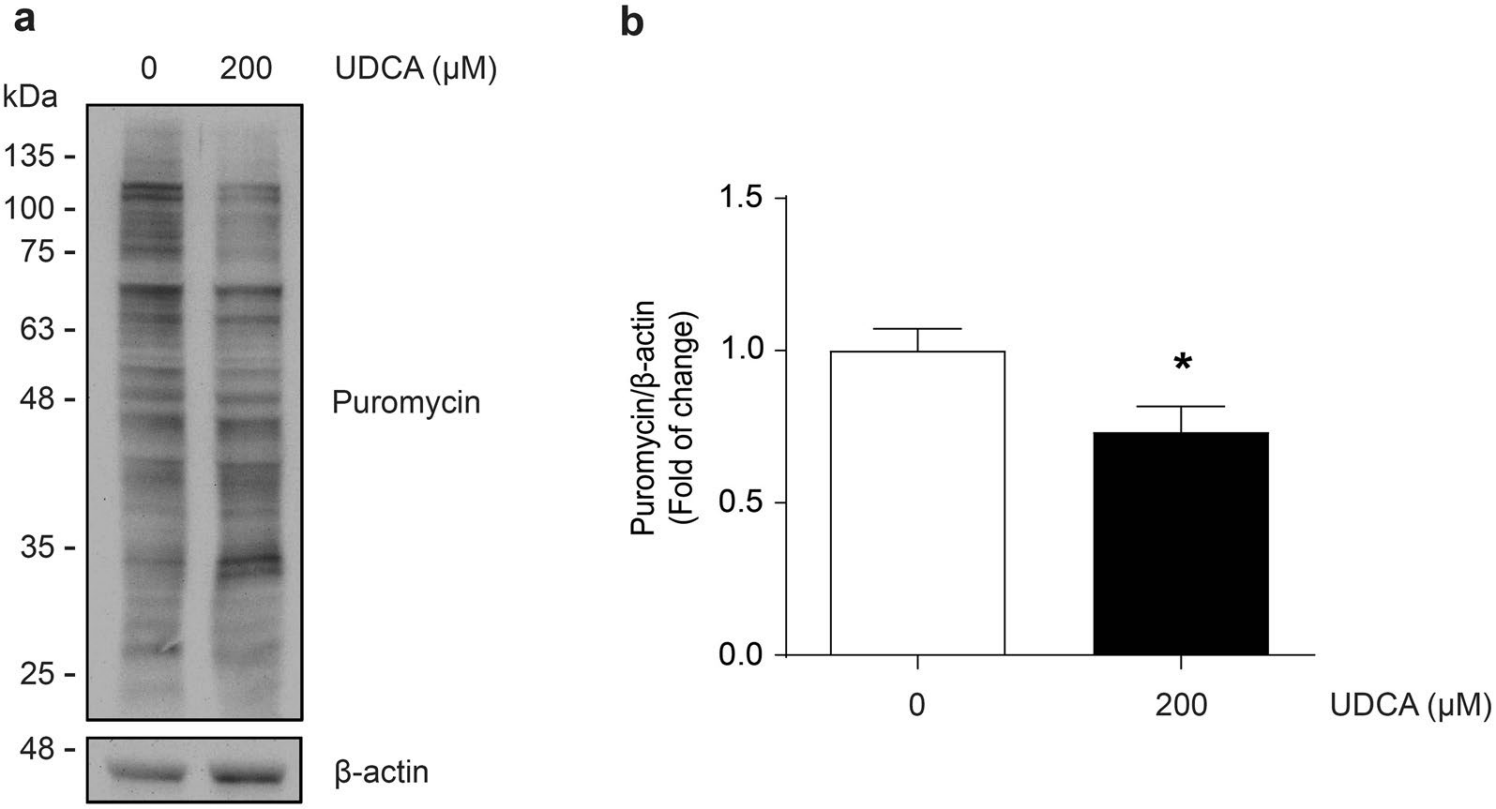
UDCA decreases puromycin incorporation into C_2_C_12_ myotubes. C_2_C_12_ myoblasts were differentiated for 4–5 days and incubated with 200 μM UDCA for 24 h. **a** Puromycin incorporation was detected by western blot analysis, using β‐actin as a loading control. Molecular weight is indicated in kDa. **b** A densitometric analysis of the puromycin incorporation bands was performed. The values are shown as a fold of change and expressed as the mean ± SD of three independent experiments (no paired t-test, *p < 0.05 with respect to the control group). *SD* standard deviation, *UDCA* ursodeoxycholic acid

**Fig. 7:**
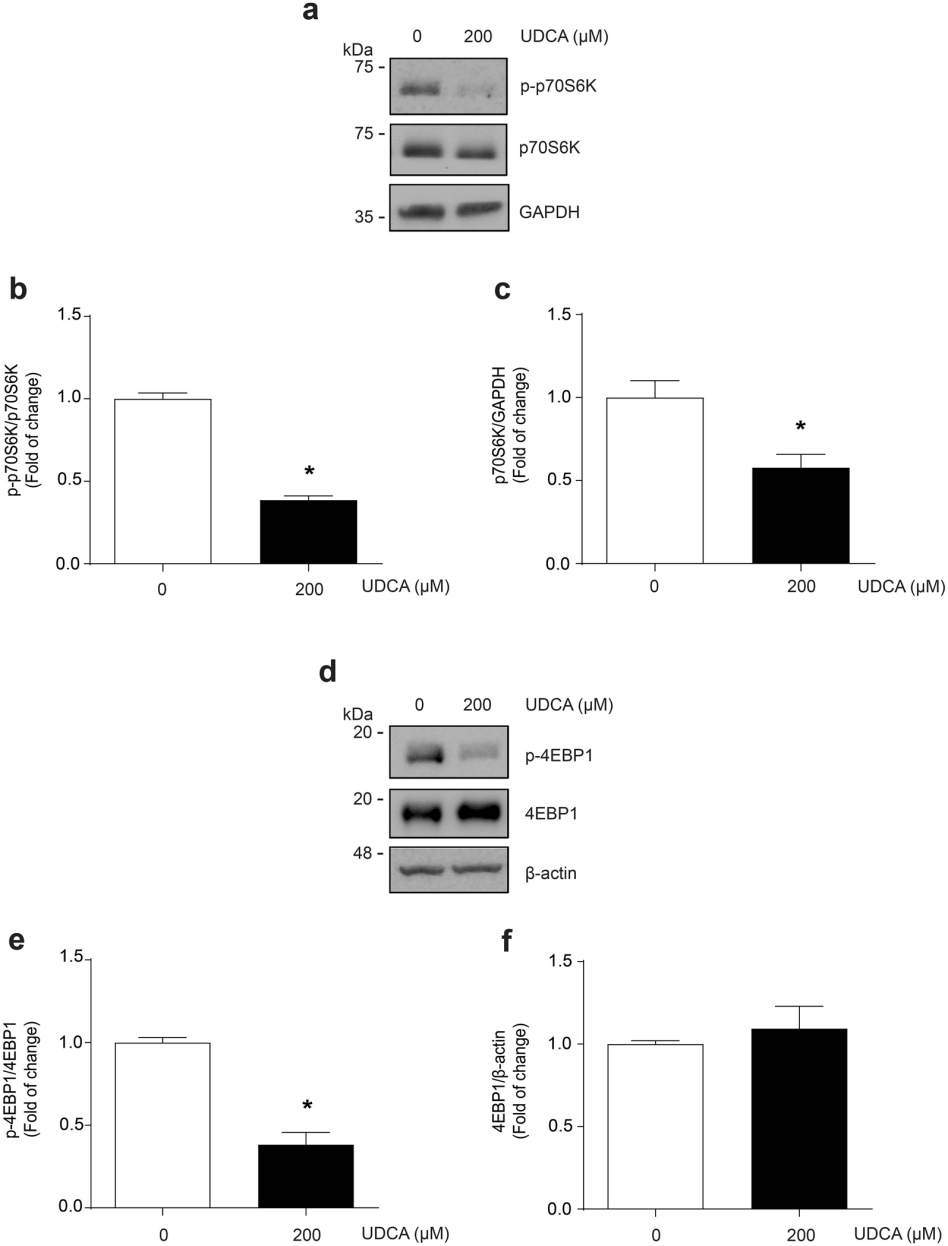
UDCA decreases p70S6K and 4EBPP1 phosphorylation and p70S6K total protein levels into C_2_C_12_ myotubes. C_2_C_12_ myoblasts differentiated for 4–5 days were incubated with 200 μM UDCA for 72 h. **a** p70S6K phosphorylation and total levels were detected by western blot analysis, using p70S6K total levels or GAPDH as a loading control, respectively. Molecular weight is indicated in kDa. **b** Densitometric analysis of p-p70S6K (Thr389) and (**c**) p70S6K total protein levels were performed. **d** 4EBP1 phosphorylation and total levels were detected by western blot analysis, using 4EBP1 total levels or β‐actin as a loading control, respectively. Molecular weight is indicated in kDa. **e** Densitometric analysis of p-4EBP1 (Ser65) and **f** 4EBP1 total protein levels was performed. The values are shown as a fold of change and expressed as the mean ± SD of three independent experiments (no paired t-test, *p < 0.05 with respect to the control group). *SD* standard deviation, *UDCA* ursodeoxycholic acid

Sarcopenia is characterized by an alteration by proteostasis, with protein synthesis and/or degradation changes [36]. Therefore, we evaluated the effect of UDCA on protein synthesis in myotubes, using the SunSET assay involving puromycin incorporation into new proteins for labeling them [37]. The pattern of labeled proteins was detected through a western blot for puromycin (Fig. 6a). The results indicate that UDCA decreased the signal of puromycin-labeled proteins at 200 μM (Fig. 6b). Further, we evaluated the status of the signaling pathways associated with protein syntheses, such as phosphorylated p70S6K (Fig. 7a) and 4EBP1 (Fig. 7d). Thus, Fig. 7b and c show the decline of the phosphorylated and total levels of p70S6K, respectively. In addition, our results show a decrease in the 4EBP1 phosphorylation (Fig. 7e) without changes in its total levels (Fig. 7f).

Together, these results suggest that UDCA decreases the rate and signaling pathways associated with protein synthesis, contributing to the sarcopenic phenotype.

### Ursodeoxycholic acid increases the LC3II/LC3I ratio with a decline in autophagic flux in myotubes

As mentioned above, the balance between protein synthesis and degradation is altered under sarcopenic conditions. UPS and autophagy are the proteolytic systems involved in sarcopenia [38]. Thus, we evaluated the participation of the atrogin-1 and MuRF-1, two E3 ubiquitin ligases associated with UPS that are regulated by transcription factor FoxO3. Neither phospho- nor total- FoxO_3_ protein levels were modified by UDCA treatment (Additional file 5a–c). MuRF-1 protein levels were unchanged (Additional file 6a and b). However, the atrogin-1 protein level was diminished by UDCA treatment in C_2_C_12_ (Additional file 6a and c).

**Fig. 8:**
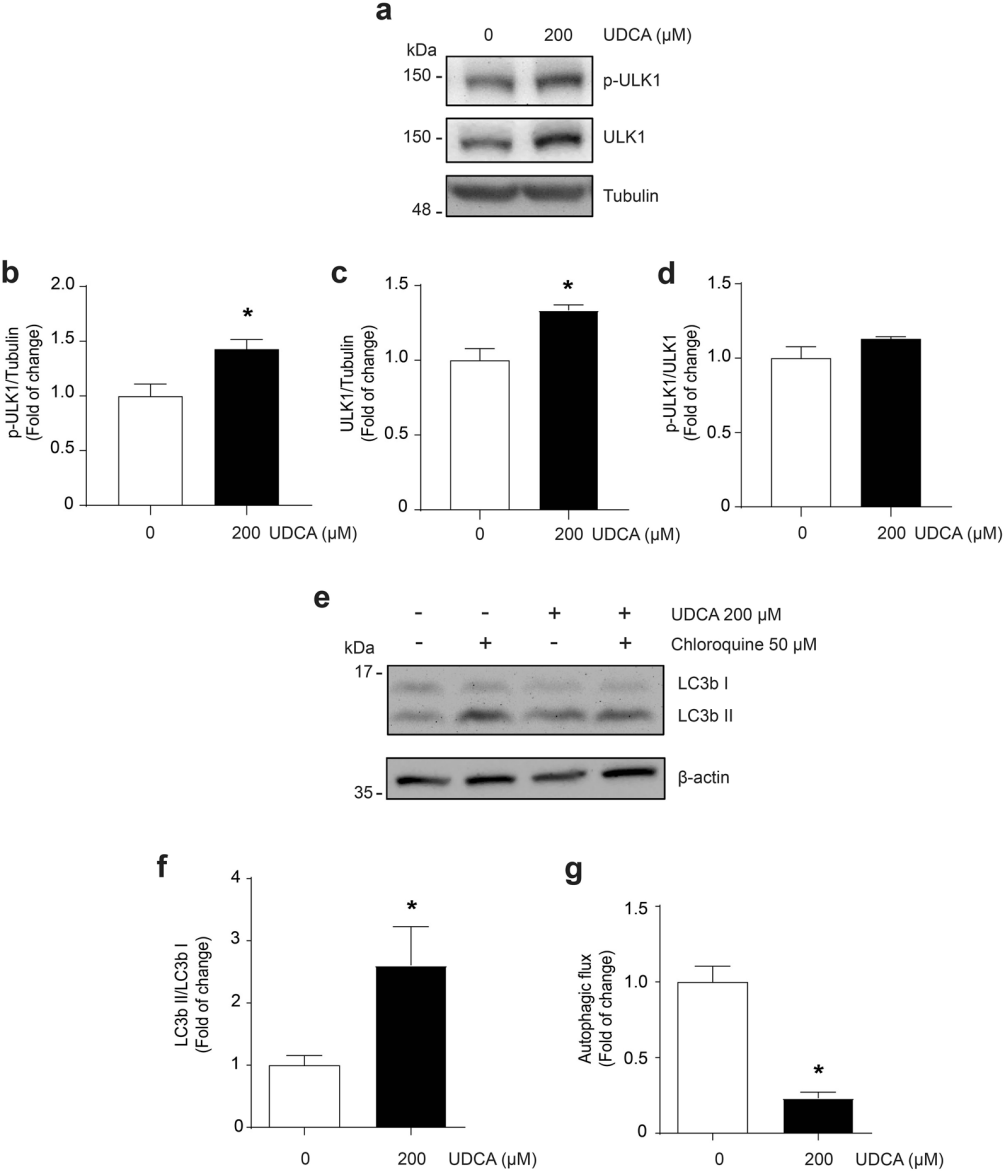
UDCA increases ULK1 levels and LC3II/LC3I ratio, and decreases autophagy flux in C_2_C_12_ myotubes. C_2_C_12_ myoblasts were differentiated for 4–5 days and incubated with 200 μM UDCA. **a** After 12 h, ULK1 phosphorylation and total levels were detected by western blot analysis, using ULK1 total levels or tubulin as a loading control, respectively. Molecular weight is indicated in kDa. **b** Densitometric analysis of p-ULK1 (Ser317) and **c** ULK1 total protein levels were performed. **d** After 72 h, LC3I and LC3II protein levels were detected by western blot analysis in the absence or presence of chloroquine. β‐actin was used as a loading control. Molecular weight is indicated in kDa. **e** A densitometric analysis of the LC3II/LC3I ratio was performed. **f** Autophagic flux was calculated using the subtraction of LC3II levels in the presence and absence of chloroquine [(LC3II + CQ) − (LC3II-CQ)]). The values are shown as a fold of change and expressed as the mean ± SD of three independent experiments (no paired t-test with Welch’s correction, *p < 0.05 with respect to the control group). *SD* standard deviation, *UDCA* ursodeoxycholic acid

Then, we evaluated the effect of UDCA on the autophagic pathway. First, we assessed a ULK1 phosphorylation associated with autophagy induction (Ser-317) [39] through western blot analysis (Fig. 8a). The results show that phospho- (Fig. 8b) and total- (Fig. 8c) ULK1 protein levels were increased by UDCA after 12 h. However, the ratio phospho-ULK1/total ULK1 was unchanged (Fig. 8d). Also, we evaluated if the effect on phospho- and total- ULK1 protein levels was maintained over time. We found no changes in either individual protein level or the phospho-ULK1/total ULK1 ratio after 24 and 48 h (Additional file 7).

Further, we evaluated the autophagic flux of myotubes. Using western blot analysis, we measured the LC3II/LC3I ratio in the absence and presence of chloroquine, an inhibitor of lysosomal H^+^-ATPase (Fig. 8e). The quantitative analysis shows that UDCA increased the LC3II/LC3I ratio without chloroquine (Fig. 8f). Moreover, the analysis of the LC3II levels in the presence and absence of chloroquine shows that UDCA decreased the autophagic flux (Fig. 8g).

**Fig. 9:**
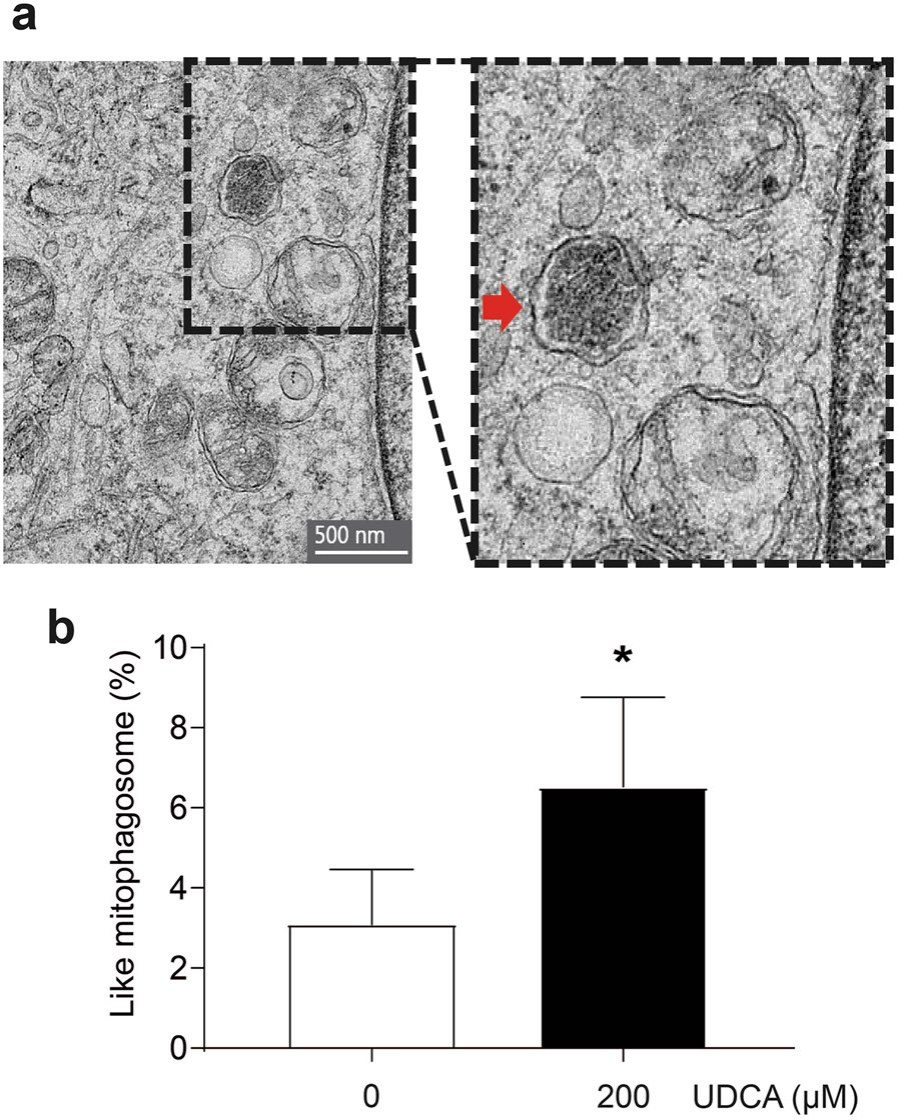
UDCA increases the presence of mitophagosome-like structures in C_2_C_12_ myotubes. C_2_C_12_ myoblasts differentiated for 4–5 days were incubated with 200 μM UDCA for 72 h. **a** Representative transmission electron microscopy images of mitophagosome-like structures in myotubes. **b** Quantification of mitophagosome-like structures number by myotube area was performed. The values are shown as a percentage of presence and expressed as the mean ± SD of four independent experiments (no paired t-test, *p < 0.05 with respect to the control group). *SD* standard deviation, *UDCA* ursodeoxycholic acid

Also, to confirm the decrease in autophagic flux induced by UDCA, we observed the mitophagosome-like structure accumulation in C_2_C_12_ cells using electron microscopy (Fig. 9a). The number of mitophagosomes was quantified manually by a microscopy expert in a blind analysis after a 72 h incubation with UDCA. It showed increased mitophagosome-like structures with respect to the control group (Fig. 9b).

These results indicate that UDCA can increase the autophagosomes by decreasing autophagy flux, contributing to the sarcopenic-like effect in skeletal cell culture. Also, the increase in ULK1 protein levels shows that UDCA modified the autophagy process, at least in the initial steps.

## Discussion

The primary treatment for the cholestatic disease is based on UDCA administration, a hydrophilic BA associated with liver and gallbladder protection [40]. However, we previously reported that other BA induced sarcopenia in mice and atrophy in isolated muscle fibers and cell culture [12, 14]. Because of its pharmacology use and molecular similarity, we analyzed the potential sarcopenic of UDCA and reported a mechanism of skeletal muscle impairment different from other BA. In mice, we demonstrated that UDCA decreased muscle mass, strength, and muscle function associated with mobility and reduced fiber diameter and sarcomeric protein after 6 weeks. Also, we validated a sarcopenic-like model with isolated muscle fibers and C_2_C_12_ cells, demonstrating in a cell culture model that skeletal muscle impairment could be related to the decline in protein synthesis and decrease of autophagic flux.

In humans, a UDCA dose within the range of 8–15 mg/kg/day has been used effectively to treat primary biliary cholangitis or gallstones [41, 42]. In this work, we used equivalent doses to those used in humans, efficiently reducing gallstones, cholestasis, and intestinal inflammation in other mice models [43–46]. In our model, UDCA administration induced sarcopenia in mice, evidencing typical alterations in patients with sarcopenia [6]. Decreased muscle strength is associated with low quality of life, hospitalization, and mortality [47, 48]. UDCA decreases strength like sarcopenia induced by chronic cholestatic liver disease [9, 12]. The hepatotoxin used in the chronic cholestatic liver disease model simulated a cholestatic illness that could be treated with UDCA [40]. Sarcopenia secondary to chronic cholestatic liver disease depends on elevated plasma BA; hence, UDCA treatment may not prevent it, owing to its sarcopenic potential.

Skeletal muscle cells are composed mainly of complex contractile protein surrounded by organelles. Consequently, the loss of synthesis–degradation protein balance impacts muscle fiber size [38]. UDCA was found to decrease muscle mass in mice and reduce the diameter of C_2_C_12_ myotubes and isolated muscle fiber. These results suggest that UDCA induces cellular adaptations that reduce the skeletal muscle cell size [49]. Our laboratory previously reported that BA affects glycolytic muscle fibers [14]. In our evaluation of an intermediate metabolism muscle, we observed the same effect in FDB-isolated fibers, confirming that UDCA diminished diameter in different muscles. Also, considering that all hindlimb muscles evaluated decreased in mass, it is expected that the effect shown in mostly fast-glycolytic muscles as TA muscle could be replicated in a slow-oxidative muscle. These changes could be associated with the diminution of sarcomeric protein levels, correlated with decreasing size, and could also be related to minor contractile capacity, affecting strength and mobility function. We demonstrated that UDCA did not alter animal motor skills, thus discarding it as a possible cause of reduced physical activity and muscle wasting [50].

Changes in the synthesis and/or degradation of proteins generate alterations in muscle mass owing to modifications of the high protein content in healthy muscle cells [51]. We demonstrated that UDCA diminishes puromycin incorporation, suggesting impairments in protein synthesis. Dysfunctional protein synthesis is also observed in other models of muscle wasting, such as immobilization and hindlimb unloading [52–56]. Since patients with cholestatic disorders present mobility problems, this could be associated with protein synthesis alteration in the skeletal muscle. Thus, UDCA administration, characterized by a decreased rate of protein synthesis as demonstrated by our results in this study, to cholestatic patients could aggravate this phenomenon, resulting in significant muscle impairment.

We analyzed the effect of UDCA administration on the signaling pathway associated with protein synthesis, evaluating p70S6K and 4EBP1 phosphorylated protein levels. Different kinases, such as mTOR, PI3K, or GSK3, can phosphorylate p70S6K [53, 57, 58]. p70S6K phosphorylation increases its kinase activity and protein synthesis rate through several mechanisms [58–60]. Decreased phosphorylated and total levels induced by UDCA can affect protein synthesis. On the other hand, the dephosphorylated 4EBP1 inhibits the cap-binding complex with eIF4F and diminishes the synthesis protein [58, 61]. It is considered that 4EBP1 is the primary molecular marker for synthesis protein inhibition [58, 62]. Our results show that UDCA diminished 4EBP1 phosphorylation. Thus, we have described the possible participation of this mechanism associated with a UDCA in contributing to sarcopenia.

Our group has observed alterations in the autophagic flux induced by CA and DCA (Tacchi, Orozco-Aguilar, et al. accepted). In the present work, we demonstrated that UDCA increased the autophagosome associated with decreased autophagic flux. The basal autophagic flux permits the degradation of protein components and dysfunctional organelles [36, 63]. It has been reported that autophagy misbalance affects skeletal muscle quality. For example, increasing autophagy flux is related to excessive cellular component degradation, whereas decreased autophagy flux is linked to the dysfunctional accumulation of organelles [64]. We confirmed the decrease of autophagic flux using the LC3 marker, and similarly, the autophagic retention showed the accumulation of mitophagosome-like structures. We recently reported that CA and DCA affected skeletal muscle through mitochondrial dysfunction, provoking alteration in mitochondrial potential and mitochondrial ROS generation [13]. Our results in the present study show evidence of mitochondrial degradation impairments, suggesting that UDCA also alters skeletal muscle function through decreased autophagy flux, accumulating dysfunctional organelles, in this work represented with mitochondria. However, a limitation in this work is the lack of evaluation of more autophagy markers that could permit a better mechanistic description of the UDCA effect. Also, UDCA increased the total levels of ULK1 but not its phosphorylation, demonstrating that UDCA altered the autophagy program. However, more studies are necessary to understand the molecular mechanism through which UDCA regulates autophagy in skeletal muscle cells. Similar effects in the detention of autophagy were observed in primary hepatic cell culture treated with CA and CDCA or in a bile duct ligature model, causing hepatocyte death [65, 66]. These results suggest that autophagy alteration could be a common effect induced by BA and a harmful event in the skeletal muscle. 

Contrary to those reported with CA and DCA, UDCA does not influence the increment of the typical E3 ubiquitin ligases, atrogin-1 and MuRF-1 [14]. Notably, UDCA diminished atrogin-1 protein levels, concomitant with decreased FoxO3 total levels in C_2_C_12_. FoxO3 nuclear translocation is associated with the transcription of atrogenes, including atrogin-1, in skeletal muscle [67–69]. Thus, UDCA-dependent changes in FoxO3 levels could be related to low levels of atrogin-1. However, considering the sarcopenic-like effect induced by UDCA, other UPS components not evaluated in this study could probably participate in protein degradation, such as other E2-E3 ligases or even the proteasome activity that previously has been used as atrophic markers in other reports [68, 70–72]. Our results confirm that UDCA affects skeletal muscle cells by a mechanism different from other hydrophobic BA. 

Our group previously reported that DCA and CA induced a sarcopenic effect in vitro and ex vivo in a TGR5-dependent mechanism [13, 14]. When evaluating the possible participation of TGR5 in the effects in muscle induced by UDCA (using SBI-115, a specific TGR5 antagonist able to inhibit the effects of BA in vitro and in vivo [73–76]), we determined that the sarcopenic-like effects derived from UDCA treatments were independent of the TGR5 activation. In 2020, the TGR5 structure, revealed by cryomicroscopy, showed a critical interaction between the position 7 hydroxyl substituent of BA and Ser247 TGR5 residue through a hydrogen bond [77]. The non-participation of TGR5 in the effects induced by UDCA in skeletal muscle cell culture could be associated with the epimerization of hydroxyl substituent that may reduce the molecular interactions with receptors. Other reports also described that UDCA is not a TGR5 agonist, showing no increase in intracellular cAMP [30, 31]. However, more analyses are necessary to elucidate the participation of another receptor than TGR5 in muscle effect dependent on UDCA. Studies of other BA have reported contrary results in skeletal muscle. LCA has hypertrophy effects; authors associate that beneficial effect with TGR5 activity [78]. However, low concentrations and short incubation periods are evaluated in those works. These antecedents suggest that BA is essential in skeletal muscle physiology and that models and concentrations used to analyze these effects are crucial to understanding this differential role. This work does not evaluate UDCA agonism. However, according to our results, the UDCA effect on sarcomeric protein levels and diameter could be mediated by another receptor different from TGR5. Among the candidates is the glucocorticoid receptor since UDCA can mediate anti-inflammatory effects via glucocorticoid receptors [58]. Considering that glucocorticoids receptor can mediate atrophic features like our results [59], sarcopenic effects dependent on UDCA could be mediated by this nuclear receptor in skeletal muscle.

UDCA has been reported to benefit skeletal muscle in chronic inflammatory models such as *mdx* mice. Specifically, this effect of UDCA is associated with NF-κB-dependent transcriptional repression [79–81]. The present study demonstrated that UDCA induced sarcopenia in adult mice and sarcopenic-like effects in skeletal muscle cells. To our knowledge, this is the first report relating to healthy mice and C_2_C_12_ cells. Importantly, UDCA showed a sarcopenic potential, like other BA, such as DCA and CA, which our group reported previously [14]. This work has tremendous clinical relevance, considering that UDCA is the first pharmacological option to treat cholestatic diseases such as primary biliary cirrhosis or sclerosing cholangitis. Our results suggest that it must be noted that collateral effects on the skeletal muscle could be similarly harmful to cholestatic conditions.

## Conclusions

In summary, this work demonstrated the sarcopenic potential of UDCA in a mouse model, confirming the harmful effect of BA on skeletal muscle. UDCA induces a cellular phenotype characterized by decreased sarcomeric protein levels and decreased diameter in C_2_C_12_ myotubes and isolated fibers. Also, we showed for the first time that UDCA could reduce protein synthesis and autophagic flux, a novel mechanism reported for a BA in skeletal muscle.

## Materials and methods

### Animals

C57BL/6J WT male mice (20–24 weeks old) were used in a randomized block experimental design. Each experimental group contained five to six mice and was treated with 200 mg/kg UDCA (Sigma-Aldrich, St. Louis, MO, USA) or vehicle (1.02% NaCl, pH 8.4) intragastrically for 6 weeks [81]. Male mice were chosen to avoid hormonal changes by reproductive cycles present in the female mice, which could affect muscle mass. The timing of UDCA administration was determined to simulate conditions of the secondary sarcopenia associated with the cholestatic disorder described previously for our group (https://doi.org/10.3390/ijms21217922). Mice were grouped in polycarbonate cages and maintained under controlled temperature, relative humidity, and photoperiod conditions. The animals were fed with a standard diet and water ad libitum. After 6 weeks, animals were euthanized, muscles were obtained, weighed, frozen in isopentane, and stored at − 80 °C until processing. Also, the tibialis anterior (TA) muscles were dissected and used for electrophysiological measurements. All animal procedures complied with international, national, and institutional animal care guidelines and were approved by the Animal Ethics Committee at the Universidad Andrés Bello Committee (approval number 012/2020).

### Muscle mass determination

After 6 weeks of treatment, animals were anesthetized (3–5% isoflurane in O_2_) for bioimpedance spectroscopy analysis (ImpediVet Vet BIS1 v.1.0.0.4) to determine corporal composition [fat-free mass (FFM), total body water (TBW), fat mass (FM)] [82].

### Strength tests

The mice were subjected to muscle strength measurement by grip strength test (Ugo Basile, Grip Strength meter 47,200) at the beginning and finalizing the experiments in the sixth week. Briefly, 15 repetitions of force were performed for the forelimb and hindlimb, and the mean was recorded in each experimental group [12]. Also, animals were evaluated using a weightlifting test. With a series of chain links of increasing length attached to a ball of tangled fine (range from 15.5 up to 54.1 g), we evaluated the weightlifting capacity, and a score was assigned. In both assays, the value of each mouse was normalized by the tibia length [10].

### Contractile measurements of isolated skeletal muscle

After euthanasia on the sixth week, the contractile properties of isolated skeletal muscle were evaluated by electrophysiology. Briefly, the removed TA was maintained in an oxygenated Krebs–Ringer solution. The muscles were stimulated with frequencies between 1 and 100 Hz for 450 ms and 2 min of rest between stimuli. The specific net force was calculated and normalized with tibia length and was expressed as mN/mm^2^ [10].

### Running test

Mice were evaluated on a treadmill tape (LE8610MTS, Panlab Harvard Apparatus, Barcelona, Spain) to determine the running capacity initially and finalize the experiments at 6 weeks. Briefly, after adaptation, animals performed an incremental speed test composed of 2 min of warm-up at 5 cm/s and increasing 2 cm/s each minute until the exhaustion of the subject [11].

### Muscle fiber diameter determination

Cryosection of 10 μm of the TA muscle was immunostained with rat anti-laminin (1:100; Santa Cruz, Dallas, TX, USA) and according to standard procedures. Images were acquired in a Motic BA310 fluorescence microscope (Motic, Hong Kong). Fiber sizes were determined by the minimal Feret diameter of each fiber was quantified by the software MyoVision (University of Kentucky, USA) [83].

### Rotarod

The mice performed an exercise on a rotarod (LE8205, Panlab Harvard Apparatus, Barcelona, Spain) at the beginning and finalizing the experiments at the sixth week, initiating with a speed of 4 rpm and gradually increasing to 30 rpm for 5 min. The total time that the animals stayed on the rod was measured [12].

### Open field exploration

The open field test consisted of observing spontaneous locomotor activity in a rectangular-polycarbonate chamber (50 cm × 30 cm × 20 cm) at the beginning and finalizing the experiments in the sixth week. The testing room was illuminated with white light and was noise-free. All animals were singly placed in the center of the chamber and video recorded for 5 min for later analyses. Locomotion (measured as distance traveled in meters) was registered using the video analysis software Kinovea 0.8.15. The apparatuses were cleaned with 70% ethanol between animals [9].

### Cell culture

The skeletal muscle cell line ATCC C_2_C_12_ was grown on DMEM 10% fetal bovine serum and differentiated on DMEM 2% horse serum until day 4 to obtain myotubes, as previously described [84]. The myotubes were incubated with ursodeoxycholic acid (UDCA) (Sigma-Aldrich, St. Louis, MO, USA) at 50, 100, 200, and 400 μM for viability test and dose dependence assay, and 200 μM for other experiments for the time indicated in each figure. Treatment times with UDCA were determined based on sarcopenic-like features induced by DCA and CA, as we previously demonstrated (https://doi.org/10.1002/jcp.29839). 

### Cell viability

The cellular viability was determined by the MTT assay. C_2_C_12_ myotubes were incubated with several concentrations of UDCA (0, 50, 100, and 200 μM) for 72 h. At the end of the experiment, 10 μl of MTT solution (5 mg/ml, pH 7.5) was added. After 1 h, blue formazan crystals were resolved with 100 μl of DMSO. Absorbance was measured at 595 nm [9].

### Western blot analysis

For the protein extracts, myotubes or tibial anterior muscles were homogenized in a radioimmunoprecipitation assay (RIPA) buffer containing phosphatases inhibitors to protect the phosphorylation status and with 1 mM of a cocktail of protease inhibitors (Sigma-Aldrich, St. Louis, MO, USA) and 1 mM of phenylmethylsulfonyl fluoride (Sigma-Aldrich, St. Louis, MO, USA). Twenty to thirty micrograms of proteins determined by Micro BCA™ Protein Assay Kit (Thermo Fisher Scientific, Waltham, MA, USA) were subjected to SDS-PAGE and transferred onto polyvinylidene difluoride membranes (Thermo Fisher Scientific, Waltham, MA, USA). The immunoblotting was developed with the following primary antibodies: mouse anti-MHC (1:1000 MF-20; Developmental Studies, Hybridoma Bank, University of Iowa, Iowa, IA, USA), mouse anti-troponin I (1:1000; Cell Signaling, Danvers, MA, USA), mouse anti-GAPDH (1:2000; Santa Cruz, Dallas, TX, USA), mouse anti-αTubulin (1:1000; Santa Cruz, Dallas, TX, USA), mouse anti-β-actin (1:2000, Abcam, Cambridge, MA, USA), mouse anti-puromycin (EMD Millipore, Burlintong, MA, USA), rabbit anti-4E-BP1 (1:1000; Cell Signaling, Danvers, MA, USA), rabbit anti-phospho-4E-BP1 (1:1000; Cell Signaling, Danvers, MA, USA), rabbit anti-p70 S6 kinase (1:1,000; Cell Signaling, Danvers, MA, USA), rabbit anti-phospho-p70 S6 kinase (1:1000; Cell Signaling, Danvers, MA, USA), rabbit anti-FOXO3A (1:1000, BIOSS, Woburn, MA, USA), rabbit anti-phospho-FOXO3A (1:1000, BIOSS, Woburn, MA, USA), rabbit anti-atrogin-1 (1:500, ECM Biosciences, Versailles, KY, USA), rabbit anti-MuRF-1 (1:500, ECM Biosciences, Versailles, KY, USA), anti-ULK1 (1:1000, Cell Signaling, Danvers, MA, USA), anti-phospho-ULK1 (1:1000, Cell Signaling, Danvers, MA, USA), anti-LC3B (1:1000, Cell Signaling, Danvers, MA, USA). Furthermore, the membranes were incubated with the respective secondary antibody [goat anti-mouse IgG-HRP (1:10,000; Santa Cruz, Dallas, TX, USA), mouse anti-rabbit IgG-HRP (1:10,000; Santa Cruz, Dallas, TX, USA)]. The immunoreaction was visualized by enhanced chemiluminescence (Thermo Scientific, Waltham, MA, USA). Images were acquired using the Fotodyne FOTO/Analyst Luminary Workstation Systems (Fisher Scientific, St. Waltham, MA, USA), and the quantification of the bands was performed utilizing densitometric analysis using ImageJ software [National Institutes of Health (NIH), Bethesda, MD, USA].

### Skeletal muscle fiber cultures

Isolated fibers from flexor digitorum brevis (FDB) muscle of C57BL/6J mice were obtained by enzymatic digestion of the whole muscle for 90 min at 37 °C with collagenase type IV (Worthington, Lakewood, NJ, USA). Afterward, the muscle was mechanically dissociated by passage through fire-polished Pasteur pipettes. Also, single extensor digitorum longus (EDL) myofibers were extracted individually using a dissecting microscope and fire‐polished pipettes and transferred serially into fresh F12–15% HS. In both cases, approximately 20–50 myofibers were transferred into 24‐well plates covered with Matrigel and incubated with UDCA 200 μM for 72 h in a humidified, 37 °C, 5% CO_2_ incubator [12, 85].

### Immunofluorescence microscopy

Myotubes, or isolated fibers, were treated with UDCA at concentrations indicated in each figure. After 72 h, the cells mentioned above were fixed in 4% paraformaldehyde overnight at 4 °C, permeabilized with 0.05% Triton X‐100 for 10 min, and blocked with 1% bovine serum albumin for 30 min. Myotubes and fibers were marked with 1:100 mouse anti‐MHC (MF‐20; Developmental Studies, Hybridoma Bank, University of Iowa) in PBS-1% BSA. After incubation and several washes with PBS-1% BSA, bound antibodies were detected with 1:250 affinity‐purified Alexa Fluor dye‐conjugated goat anti‐mouse antibody (Life Technologies). Also, the samples were marked with 1 μg/ml Hoechst 33,258 for 10 min. The sections were mounted with a fluorescent dye‐stained Fluoromont™ Aqueous Mounting Medium (Sigma-Aldrich, St. Louis, MO, USA) under glass coverslips. Samples were observed on a Motic BA310 epifluorescence microscope (Motic, Hong Kong). Photographs obtained were analyzed (ImageJ, NIH, Bethesda, MD, USA), and the minimal Feret diameters were measured in approximately 80 myotubes from 10 random fields and in 20–50 fibers from each treatment [14].

### SUrface SEnsing of Translation (SUnSET) assay

The protein levels of puromycin residues were detected by Western blot. The incorporation rate was used as a parameter of protein synthesis on treated myotubes with UDCA 200 μM for 72 h. For puromycin incorporation, cells were incubated for 24 h with 1 mM puromycin before cells were harvested in homogenization buffer for Western blot [86].

### Autophagy assay

The protein levels of LC3I and LC3II were detected by Western blot. The LC3II/LC3I ratio was analyzed as a parameter of autophagosome formation on treated myotubes with UDCA 200 μM for 72 h. Besides, autophagic flux was determined by analysis of the difference between LC3II protein levels in cells incubated in the presence and absence of 50 mM of chloroquine (CQ) (Sigma-Aldrich, St Louis, MO, USA) [87].

### Transmission electron microscopy

Myotubes were treated with UDCA 200 μM for 72 h. After, the cells were fixed in glutaraldehyde 4%. Washes were made with sodium cacodylate buffer 0.1 M before staining osmium tetroxide 2% in the same buffer for 2 h. Another staining was done with uranyl acetate 1% for 2 h after washes with cacodylate buffer. The myotube samples were dehydrated with acetone gradients and embedded in Epon [88, 89]. Finally, 80-nm sections were cut and mounted on electron microscopy grids for examination using a transmission electron microscope (Philips, Tecnai 12 at 80 kV). The number of mitophagosome-like structures was counted manually by a blind investigator at 20,000 × magnification.

### Statistics

The assumption of normality distribution was tested using the Kolmogorov–Smirnov test and differences in standard deviations with Brown-Forsythe test. Data in the text and tables are presented as the mean ± standard error of the mean (SEM). The box-and-whisker graphs show the median (the horizontal line across the box), the 25th and 75th quartiles (the lower and upper box lines, respectively), and the mean (the + symbol inside the box). All statistical analyses were performed with Prism 8.0 analysis software (GraphPad Software, San Diego, CA, USA). Viability and dose dependence assays were analyzed using a one-way analysis of variance (ANOVA). The Bonferroni post hoc test adjusted multiple comparisons or Kruskal–Wallis (non-parametric analysis), detailed in the legend of each figure. Furthermore, animal experiments, fiber assays, and molecular pathways tests were statistically analyzed with a t-test or t-test with Welch’s correction when there is no homoscedasticity. The p-values < 0.05 were considered statistically significant.

## Supplementary Information

**Additional file 1.** Motor skills were maintained in mice treated with UDCA after 6 weeks. C57BL/6 male mice were treated orally with UDCA 200 mg/kg corporal body weight for 6 weeks (1.04% NaCl pH 8.4 to the control group). Mice were monitored daily, and muscular evaluations were made when finishing the treatment. (a) An open field test was recorded to calculate the distance traveled in 5 min. Values represent the distance reached by the animal in the sixth week. (b) The rotarod test was performed at the end of treatment. Values represent the time on the rod during 5 min of the test. The result shows the individual value of the subject, with the mean ± SEM for each group (n = 5 mice per group, no paired t-test). UDCA, ursodeoxycholic acid; SEM, standard error of the mean.

**Additional file 2.** UDCA decreased the fiber diameter in soleus muscles. C57BL/6 male mice were treated with UDCA 200 mg/kg corporal body weight for 6 weeks orally (1.04% NaCl pH 8.4 to control group). Mice were monitored daily, and muscular evaluations were made at the beginning and during the sixth week. (a) Soleus (SOL) muscle cross-sections were stained with laminin to delimit the sarcolemma. The scale bar indicates 100 μm. (b) The minimal Feret’s diameters were calculated using the MyoVision software. Fiber diameters were grouped from 5 to 80 μm to quantify the total fiber percentage by each group. (c) Accumulative frequency analysis to UDCA and control group were plotted. (d) The area under the curve was calculated in accumulative frequency to fiber diameters using GraphPad Prism 8.0 software. The result shows the mean ± SEM for each group. (n = 5 mice per group, no paired t-test, *p < 0.05 with respect to the control group). SEM, standard error of the mean; UDCA, ursodeoxycholic acid.

**Additional file 3.** UDCA does not alter the cell viability in C_2_C_12_ myotubes. C_2_C_12_ myoblasts were differentiated for 4–5 days and then set with 0, 50, 100, 200, and 400 μM UDCA for (a) 48 h or (b) 72 h. Cell viability was evaluated through the MTT test. The values indicate the percentage of the viable cells, expressed as the mean ± SD of three independent experiments in duplicate (one‐way ANOVA, post‐hoc Dunnet, *p < 0.05 with respect to the control). ANOVA, analysis of variance; UDCA, ursodeoxycholic acid; MTT, 3-(4,5-dimethylthiazol-2-yl)-2,5-diphenyltetrazolium bromide; SD, standard deviation.

**Additional file 4.** Antagonism of the TGR5 receptor does not prevent the reduction of the diameter and troponin I level in C_2_C_12_ myotubes induced by UDCA. Myotubes were incubated with 200 μM UDCA in the absence or presence of 10 μM SIB-115 for 72 h. (a) MHC was detected by indirect immunofluorescence and used to delimit the myotube diameter. Images were captured by fluorescence microscopy. The scale bar indicates 100 μm. (b) The diameter of the myotubes was measured using ImageJ software. The quantification was performed, and the individual values for the myotube were plotted (Kruskal—Wallis, *p < 0.05 with respect to the control). (c) Protein levels of troponin I were determined by western blot analysis, using GAPDH as a loading control. Molecular weight is indicated in kDa. (d) Densitometric analysis of the troponin I bands was performed. The values are shown as a fold of change and expressed as the mean ± SD of three independent experiments (one‐way ANOVA, post‐hoc Bonferroni, *p < 0.05 with respect to the control). ANOVA, analysis of variance; UDCA, ursodeoxycholic acid; MHC, myosin heavy chain; SD, standard deviation.

**Additional file 5.** UDCA decreases FoxO3 total protein levels in C_2_C_12_ myotubes. C_2_C_12_ myoblasts differentiated for 4–5 days were incubated with 200 μM UDCA for (a) 12 h, (b) 24 h, and (c) 48 h. FoxO3 phosphorylation and total levels were detected by western blot analysis, using FoxO3 total levels or tubulin as a loading control, respectively. Molecular weight is indicated in kDa. Densitometric analysis of p-FoxO3 (Ser253) and FoxO3 total protein levels was performed. The values are shown as a fold of change and expressed as the mean ± SD of three independent experiments (no paired t-test, *p < 0.05 with respect to the control group). SD, standard deviation; UDCA, ursodeoxycholic acid.

**Additional file 6.** UDCA decreases atrogin-1 levels in C_2_C_12_ myotubes. C_2_C_12_ myoblasts differentiated for 4–5 days were incubated with 200 μM UDCA for 72 h. (a) MuRF-1 and atrogin-1 levels were detected by western blot analysis, using GAPDH as a loading control, respectively. Molecular weight is indicated in kDa. (b) Densitometric analysis of MuRF-1 and (c) atrogin-1 was performed. The values are shown as a fold of change and expressed as the mean ± SD of three independent experiments (no paired t-test, *p < 0.05 with respect to the control group). SD, standard deviation; UDCA, ursodeoxycholic acid.

**Additional file 7.** UDCA does not modify ULK1 phosphorylation or total protein levels into C_2_C_12_ myotubes. C_2_C_12_ myoblasts differentiated for 4–5 days were incubated with 200 μM UDCA for (a) 24 h and (b) 48 h. ULK1 phosphorylation and total levels were detected by western blot analysis, using ULK1 total levels or tubulin as a loading control, respectively. Molecular weight is indicated in kDa. Densitometric analysis of p-ULK1 (Ser317) and ULK1 total protein levels was performed. The values are shown as a fold of change and expressed as the mean ± SD of three independent experiments (no paired t-test). SD, standard deviation; UDCA, ursodeoxycholic acid.

## Data Availability

The datasets during and/or analyzed during the current study are available from the corresponding author upon reasonable request.

## Abbreviations

4EBP-1: Eukaryotic translation initiation factor 4E-binding protein 1
AUC: Area under of curve
BA: Bile acids
BSA: Bovine serum albumin
CA: Cholic acid
CCLD: Chronic cholestatic liver disease
CQ: Chloroquine
DCA: Deoxycholic acid
DIA: Diaphragm
DMEM: Dulbecco’s Modified Eagle Medium
EDL: Extensor digitorum longus
FDB: Flexor digitorum brevis
FFM: Fat-free mass
FM: Fat mass
GAST: Gastrocnemius
LC3: Microtubule-associated protein 1A/1B-light chain 3
MHC: Myosin heavy chain
mTOR: Mammalian target of rapamycin
MuRF-1: Muscle RING-finger protein-1
NAFLD: Nonalcoholic fatty liver disease
NF-κB: Factor nuclear kappa B
p70S6K: 70-KDa ribosomal protein S6 kinase
RIPA: Radioimmunoprecipitation assay buffer
ROS: Reactive oxygen species
SOL: Soleus
SUNSET: SUrface SEnsing of Translation assay
TA: Tibilais anterior
TBW: Total body water
UDCA: Ursodeoxycholic acid
ULK1: Unc-51 like autophagy activating kinase
UPS: Ubiquitin–proteasome system
