# Supplementary figures and images for: Ursodeoxycholic acid induces sarcopenia associated with decreased protein synthesis and autophagic flux
Source: Biol Res. 2023 May 27;56:28. doi: 10.1186/s40659-023-00431-8 (PMC10224307; doi:10.1186/s40659-023-00431-8)

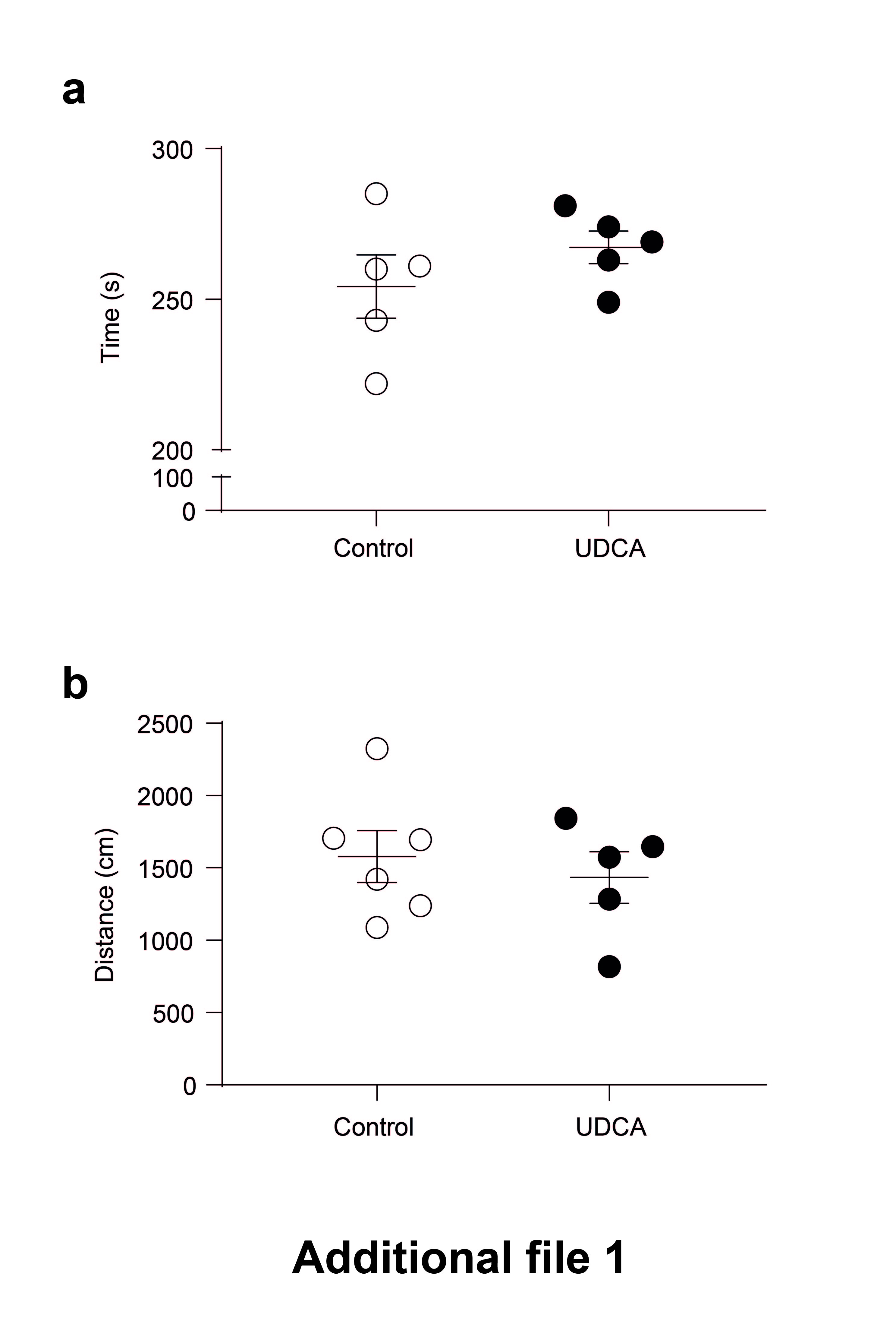

Supplement: Supplementary file 1 — Additional file 1. Motor skills were maintained in mice treated with UDCA after 6 weeks. C57BL/6 male mice were treated orally with UDCA 200 mg/kg corporal body weight for 6 weeks (1.04% NaCl pH 8.4 to the control group). Mice were monitored daily, and muscular evaluations were made when finishing the treatment. (a) An open field test was recorded to calculate the distance traveled in 5 min. Values represent the distance reached by the animal in the sixth week. (b) The rotarod test was performed at the end of treatment. Values represent the time on the rod during 5 min of the test. The result shows the individual value of the subject, with the mean ± SEM for each group (n = 5 mice per group, no paired t-test). UDCA, ursodeoxycholic acid; SEM, standard error of the mean. [file 40659_2023_431_MOESM1_ESM.jpg]

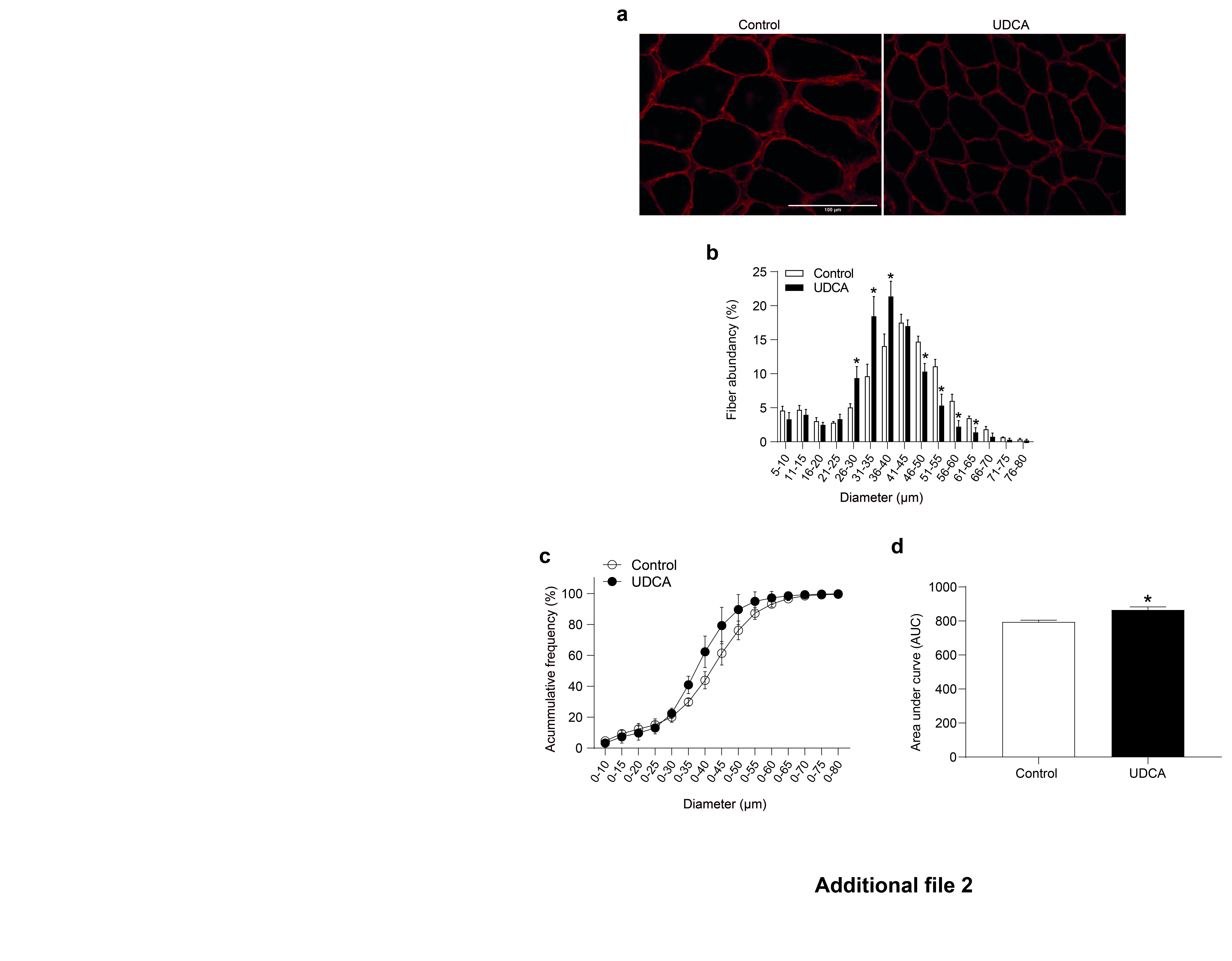

Supplement: Supplementary file 2 — Additional file 2. UDCA decreased the fiber diameter in soleus muscles. C57BL/6 male mice were treated with UDCA 200 mg/kg corporal body weight for 6 weeks orally (1.04% NaCl pH 8.4 to control group). Mice were monitored daily, and muscular evaluations were made at the beginning and during the sixth week. (a) Soleus (SOL) muscle cross-sections were stained with laminin to delimit the sarcolemma. The scale bar indicates 100 μm. (b) The minimal Feret’s diameters were calculated using the MyoVision software. Fiber diameters were grouped from 5 to 80 μm to quantify the total fiber percentage by each group. (c) Accumulative frequency analysis to UDCA and control group were plotted. (d) The area under the curve was calculated in accumulative frequency to fiber diameters using GraphPad Prism 8.0 software. The result shows the mean ± SEM for each group. (n = 5 mice per group, no paired t-test, *p < 0.05 with respect to the control group). SEM, standard error of the mean; UDCA, ursodeoxycholic acid. [file 40659_2023_431_MOESM2_ESM.jpg]

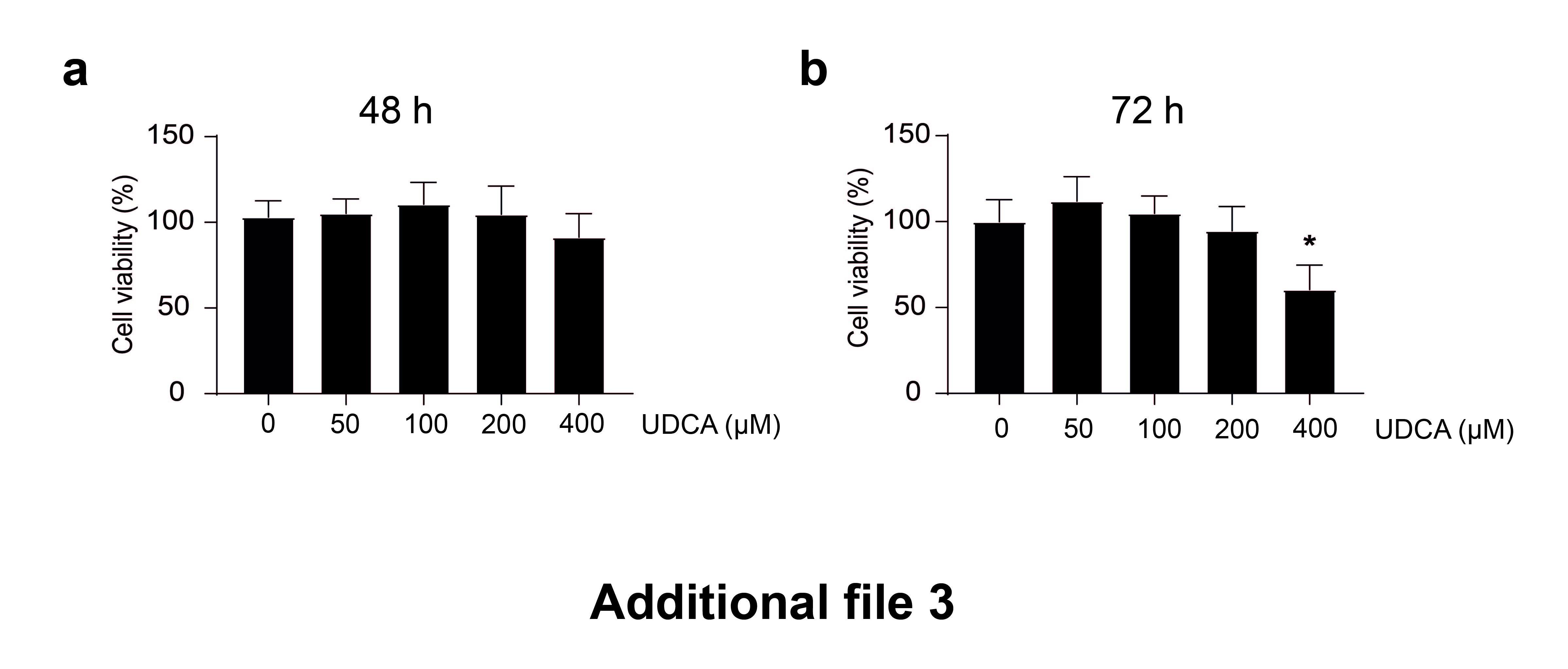

Supplement: Supplementary file 3 — Additional file 3. UDCA does not alter the cell viability in C2C12 myotubes. C2C12 myoblasts were differentiated for 4–5 days and then set with 0, 50, 100, 200, and 400 μM UDCA for (a) 48 h or (b) 72 h. Cell viability was evaluated through the MTT test. The values indicate the percentage of the viable cells, expressed as the mean ± SD of three independent experiments in duplicate (one‐way ANOVA, post‐hoc Dunnet, *p < 0.05 with respect to the control). ANOVA, analysis of variance; UDCA, ursodeoxycholic acid; MTT, 3-(4,5-dimethylthiazol-2-yl)-2,5-diphenyltetrazolium bromide; SD, standard deviation. [file 40659_2023_431_MOESM3_ESM.jpg]

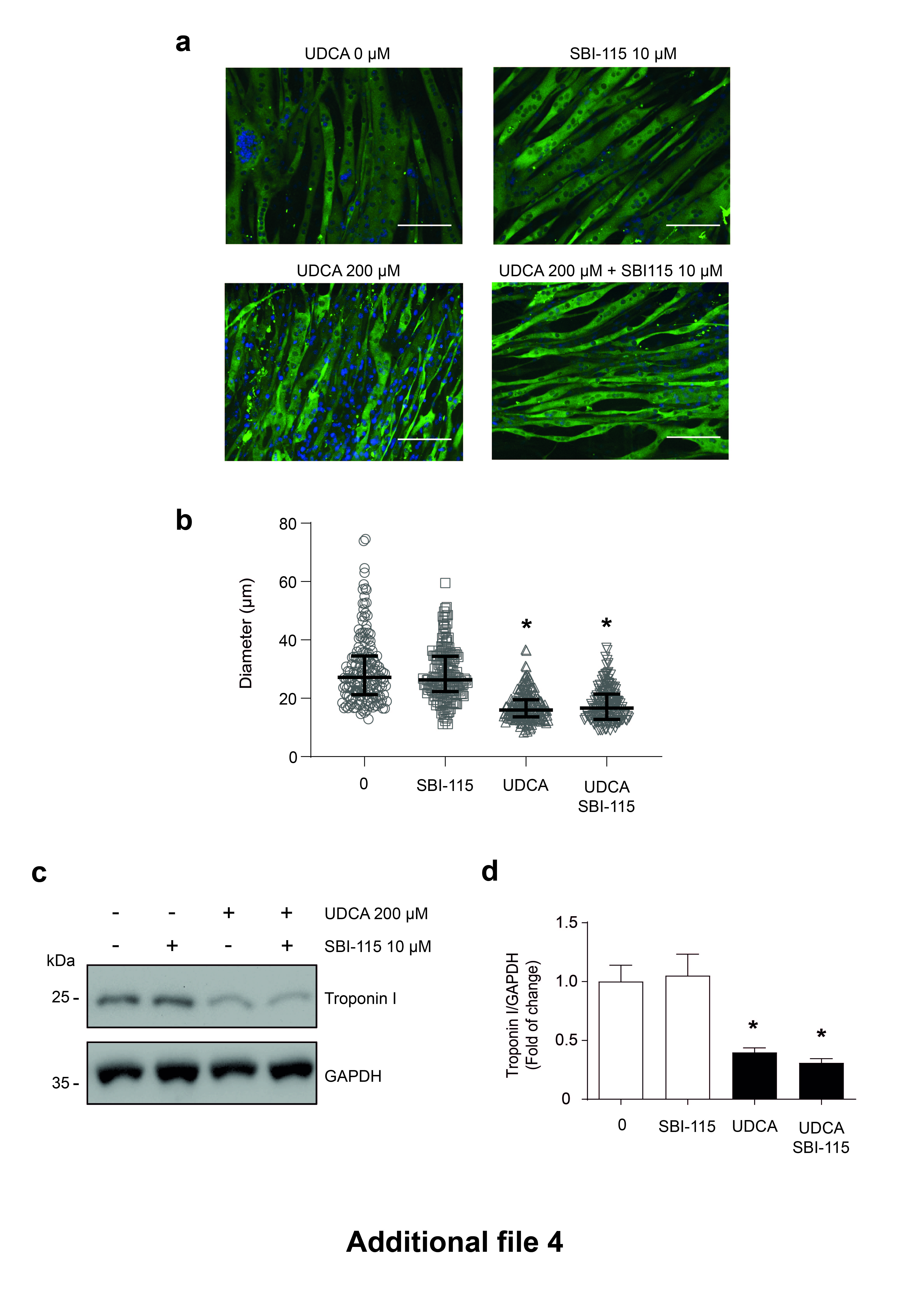

Supplement: Supplementary file 4 — Additional file 4. Antagonism of the TGR5 receptor does not prevent the reduction of the diameter and troponin I level in C2C12 myotubes induced by UDCA. Myotubes were incubated with 200 μM UDCA in the absence or presence of 10 μM SIB-115 for 72 h. (a) MHC was detected by indirect immunofluorescence and used to delimit the myotube diameter. Images were captured by fluorescence microscopy. The scale bar indicates 100 μm. (b) The diameter of the myotubes was measured using ImageJ software. The quantification was performed, and the individual values for the myotube were plotted (Kruskal—Wallis, *p < 0.05 with respect to the control). (c) Protein levels of troponin I were determined by western blot analysis, using GAPDH as a loading control. Molecular weight is indicated in kDa. (d) Densitometric analysis of the troponin I bands was performed. The values are shown as a fold of change and expressed as the mean ± SD of three independent experiments (one‐way ANOVA, post‐hoc Bonferroni, *p < 0.05 with respect to the control). ANOVA, analysis of variance; UDCA, ursodeoxycholic acid; MHC, myosin heavy chain; SD, standard deviation. [file 40659_2023_431_MOESM4_ESM.jpg]

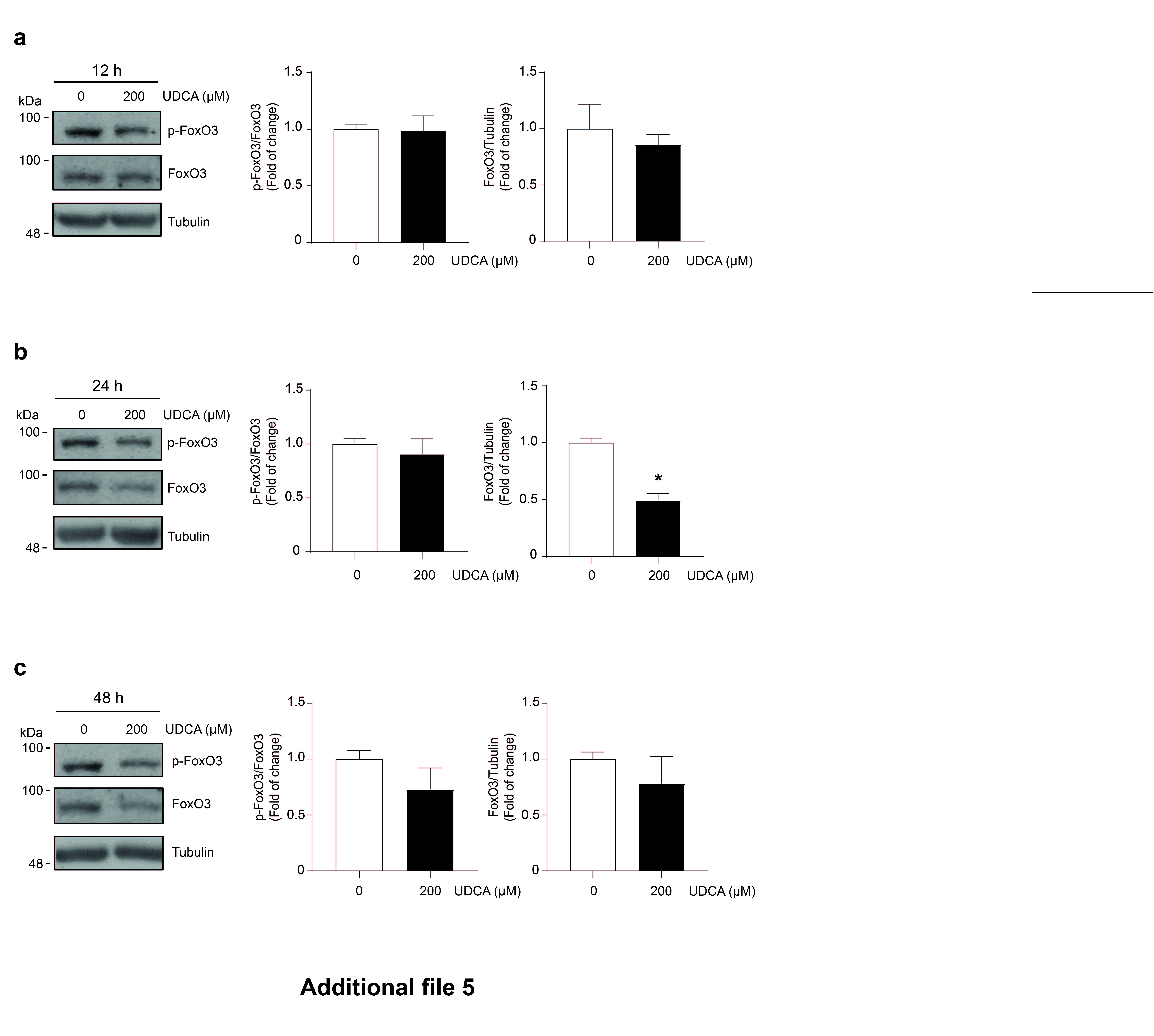

Supplement: Supplementary file 5 — Additional file 5. UDCA decreases FoxO3 total protein levels in C2C12 myotubes. C2C12 myoblasts differentiated for 4–5 days were incubated with 200 μM UDCA for (a) 12 h, (b) 24 h, and (c) 48 h. FoxO3 phosphorylation and total levels were detected by western blot analysis, using FoxO3 total levels or tubulin as a loading control, respectively. Molecular weight is indicated in kDa. Densitometric analysis of p-FoxO3 (Ser253) and FoxO3 total protein levels was performed. The values are shown as a fold of change and expressed as the mean ± SD of three independent experiments (no paired t-test, *p < 0.05 with respect to the control group). SD, standard deviation; UDCA, ursodeoxycholic acid. [file 40659_2023_431_MOESM5_ESM.jpg]

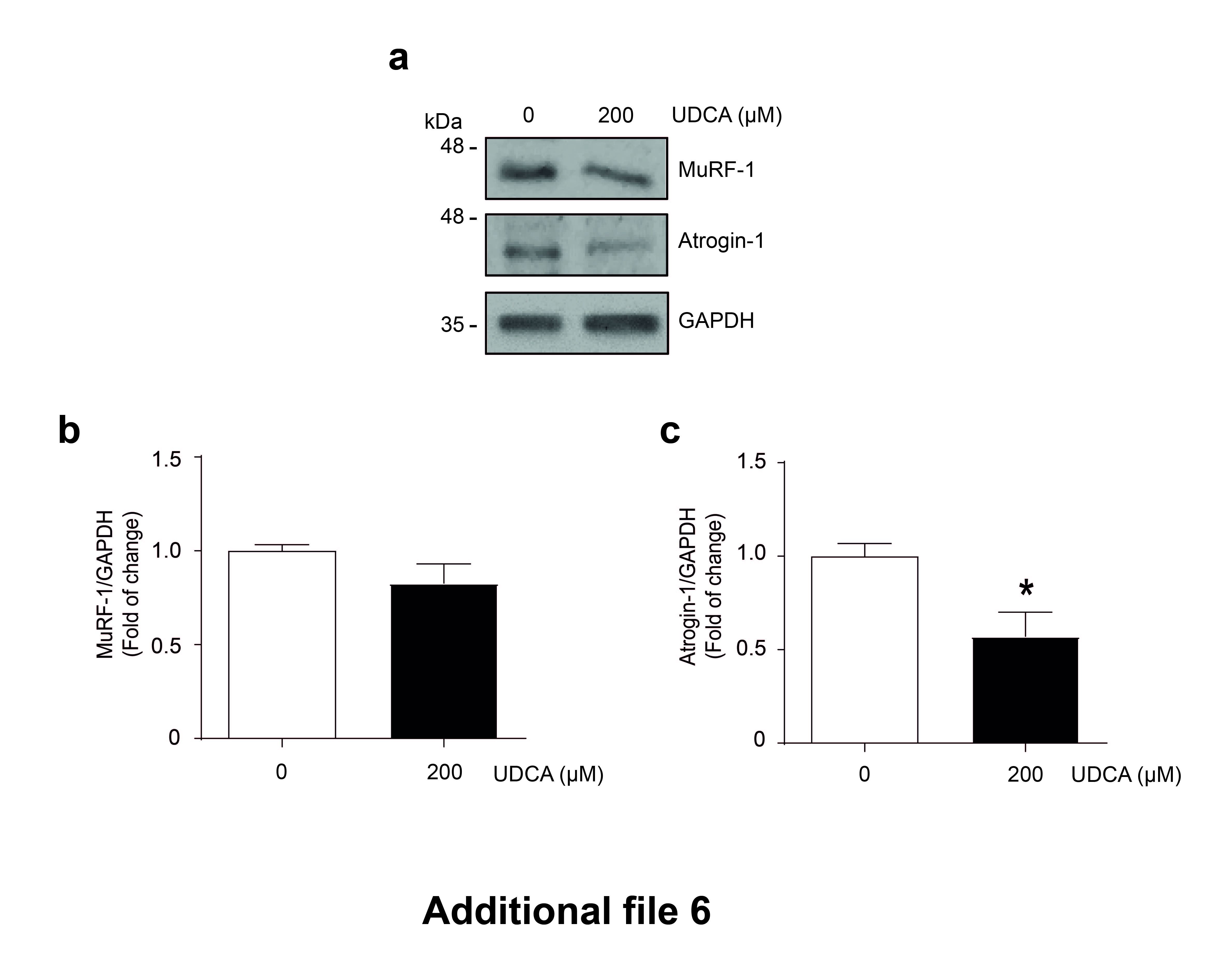

Supplement: Supplementary file 6 — Additional file 6. UDCA decreases atrogin-1 levels in C2C12 myotubes. C2C12 myoblasts differentiated for 4–5 days were incubated with 200 μM UDCA for 72 h. (a) MuRF-1 and atrogin-1 levels were detected by western blot analysis, using GAPDH as a loading control, respectively. Molecular weight is indicated in kDa. (b) Densitometric analysis of MuRF-1 and (c) atrogin-1 was performed. The values are shown as a fold of change and expressed as the mean ± SD of three independent experiments (no paired t-test, *p < 0.05 with respect to the control group). SD, standard deviation; UDCA, ursodeoxycholic acid. [file 40659_2023_431_MOESM6_ESM.jpg]

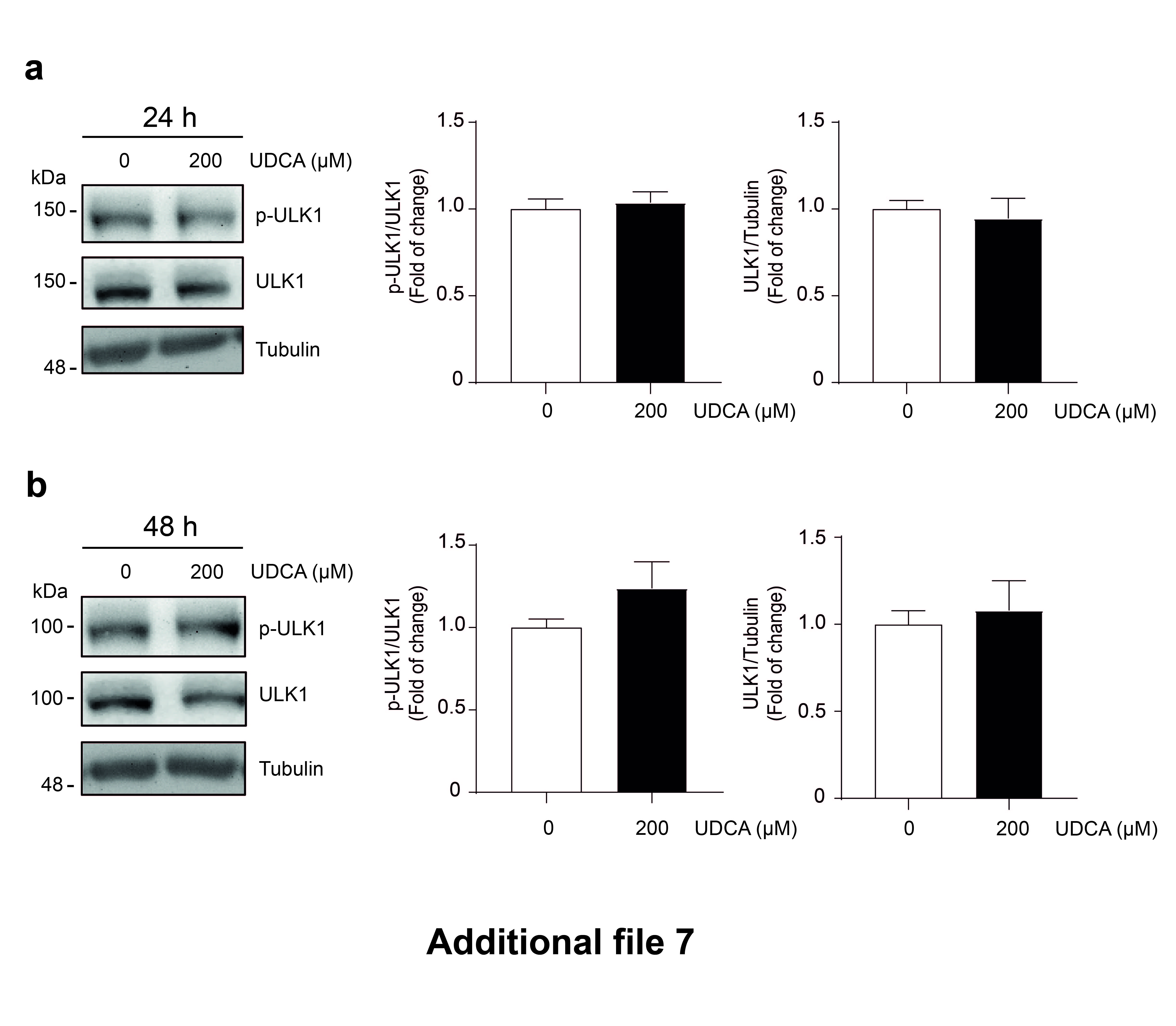

Supplement: Supplementary file 7 — Additional file 7. UDCA does not modify ULK1 phosphorylation or total protein levels into C2C12 myotubes. C2C12 myoblasts differentiated for 4–5 days were incubated with 200 μM UDCA for (a) 24 h and (b) 48 h. ULK1 phosphorylation and total levels were detected by western blot analysis, using ULK1 total levels or tubulin as a loading control, respectively. Molecular weight is indicated in kDa. Densitometric analysis of p-ULK1 (Ser317) and ULK1 total protein levels was performed. The values are shown as a fold of change and expressed as the mean ± SD of three independent experiments (no paired t-test). SD, standard deviation; UDCA, ursodeoxycholic acid. [file 40659_2023_431_MOESM7_ESM.jpg]
